# Nematicidal and Insecticidal Compounds from the Laurel Forest Endophytic Fungus *Phyllosticta* sp.

**DOI:** 10.3390/molecules29194568

**Published:** 2024-09-26

**Authors:** Carmen E. Díaz, María Fe Andrés, Patricia Bolaños, Azucena González-Coloma

**Affiliations:** 1 Instituto de Productos Naturales y Agrobiología, CSIC, Avda. Astrofísico F. Sánchez 3, 38206 La Laguna, Spain; ateneabg7@gmail.com; 2 Instituto de Ciencias Agrarias, CSIC, 28006 Madrid, Spain; mafay@ica.csic.es

**Keywords:** *Phyllosticta* sp., dioxolanones, metguignardic acid, meroterpenes, 14-*epi*-guignardone I, nematicidal activity, insecticidal activity

## Abstract

The search for natural product-based biopesticides from endophytic fungi is an effective tool to find new solutions. In this study, we studied a pre-selected fungal endophyte, isolate YCC4, from the paleoendemism *Persea indica*, along with compounds present in the extract and the identification of the insect antifeedant and nematicidal ones. The endophyte YCC4 was identified as *Phyllosticta* sp. by molecular analysis. The insect antifeedant activity was tested by choice bioassays against *Spodoptera littoralis*, *Myzus persicae,* and *Rhopalosiphum padi,* and the in vitro and in vivo mortality was tested against the root-knot nematode *Meloidogyne javanica*. Since the extract was an effective insect antifeedant, a strong nematicidal, and lacked phytotoxicity on tomato plants, a comprehensive chemical study was carried out. Two new metabolites, metguignardic acid (**4**) and (-)-*epi*-guignardone I (**14**), were identified along the known dioxolanones guignardic acid (**1**), ethyl guignardate (**3**), guignardianones A (**5**), C (**2**), D (**7**), and E (**6**), phenguignardic acid methyl ester (**8**), the meroterpenes guignardone A (**9**) and B (**10**), guignarenone B (**11**) and C (**12**), (-)-guignardone I (**13**), and phyllomeroterpenoid B (**15**). Among these compounds, **1** and **4** were effective antifeedants against *S. littoralis* and *M. persicae*, while **2** was only active on the aphid *M. persicae*. The nematicidal compounds were **4**, **7**, and **8**. This is the first report on the insect antifeedant or nematicidal effects of these dioxolanone-type compounds. Since the insect antifeedant and nematicidal activity of the *Phyllosticta* sp. extract depend on the presence of dioxolanone components, future fermentation optimizations are needed to promote the biosynthesis of these compounds instead of meroterpenes.

## 1. Introduction

Endophytic fungi are capable of producing a diverse range of bioactive compounds that exhibit a wide array of biological activities, including insecticidal, antioxidant, antifungal, antiviral, antibacterial, and cytotoxic properties. The secretion of these secondary metabolites by endophytic fungi is known to host plant defense response, enabling it to better cope with both biotic and abiotic stressors [1]. Thus, endophytic fungi represent a promising novel source for the biotechnological bioproduction of valuable active compounds [2].

The Macaronesian laurel forest is an evergreen humid plant community composed of plants recruited from European/Mediterranean and tropical regions and modified due to temperature changes during the Pleistocene [3]. Laurel forest species host endophytic fungi communities common to tropical forests (*Colletotrichum*, *Pestalotiopsis*, and *Guignardia*), making *G. mangiferae*, *Glomerella acutata*, *Neofusicoccum parvum,* and *Phomopsis *sp. the dominant endophytes [4].

*Persea indica*, a perennial tree belonging to the Lauraceae family, is one of the dominant species of the Macaronesian laurel forest. *P. indica* contains ryanodane and isoryanodane diterpenes, alkene-γ-lactones, and avocadofuranes in the aerial parts. The ryanodane diterpenes are strong insect antifeedants [5,6]. Some endophytic fungi can produce secondary metabolites previously found in their host plant. For example, endophytes of *Panax sokpayensis* produce a bioactive ginsenoside found in the host plant [7], or endophytic fungi isolated from the leaf of *Catharanthus roseus* (*Alternaria alternata*, *Curvularia lunata*, *Aspergillus terrus*, and *A. clavatonanicus*) that produce anhydrovinblastine, a precursor of vinblastine, and the intermediate compounds of the biosynthetic pathway [8]. Therefore, the chemistry of the host plant could be a selection criterion for the isolation of fungal endophytes, among others.

Additionally, an extract from the endophyte identified as *G. manguiferae* (synonym: *Phyllosticta* sp.), previously isolated from *P. indica*, showed strong insect antifeedant effects against *S. littoralis* and nematicidal activity against root-knot nematode *Meloidogyne javanica* [4]. However, the active compounds were not identified. The genus *Phyllosticta* (Ascomycetes) are a group of microscopic fungi that can colonize a variety of plant hosts, including several *Citrus* species. Some *Phyllosticta* species have the capacity to cause disease, including leaf spots and black spots on fruits, while others have only been observed as endophytes [9]. *Phyllosticta capitalensis* is an endophyte and weak plant pathogen with a worldwide distribution presently known from 70 plant families [10]. *Phyllosticta* species are potential biocontrol agents. For example, *P. capitalensis* isolated from *Camellia sinensis* showed antifungal effects in dual growth tests against *Fusarium* sp., *Pestalotiosis* sp., and *Sclerotinia sclerotiorum* [11]. Furthermore, endophytic *Phyllosticta* sp. isolated from cucumber showed potential as a seed treatment agent for *Meloidogyne incognita* biocontrol [12]. 

In this work, the endophytic fungal strain YCC4 has been isolated from the leaves of *P. indica* and identified as *Phyllosticta* sp. based on molecular techniques. The fermentation of YCC-4 on Czapek-Dox-Yeast liquid medium gave an ethyl acetate (EtOAc) extract with plant protection properties. The bioassay-guided fractionation of this extract led to the isolation of five active compounds (**1**, **2**, **4**, **7**, **8**) that have been identified based on their spectroscopic data. The extract and pure compounds were tested against insect pests (*Spodoptera littoralis*, *Myzus persicae*, and *Rhopalosiphum padi*) and the plant parasitic nematode *M. javanica*. Detailed findings are presented in this paper.

## 2. Results

The endophytic fungus YCC4 was isolated from leaves of the endemic Macaronesian species *P. indica* and identified as *Phyllosticta* sp. (synonym: *Guignardia* sp.). The EtOAc extract obtained from the fermentation of YCC4 was tested against insect pests (*S. littoralis*, *M. persicae*) and the root-knot nematode *M. javanica.*

The extract showed a strong antifeedant effect against *S. littoralis* (EC_50_ = 9.77 μg/cm^2^), followed by *M. persicae* (EC_50_ = 17.2 μg/cm^2^), and it was not active on *R. padi*. The nematicidal effects of the extract against *M. javanica* J2 were very strong (24–72 h mortality range of 96–100%), with an effective LC_50_ concentration of 0.44 mg/mL at 72 h. Furthermore, the extract also had effective egg hatching inhibition effects (64% inhibition with respect to the control after 28 days) (Table 1). The extract was also tested for phytotoxic effects on seeds of mono- and dicotyledoneous plant species (*Lolium perenne*, *Lactuca sativa,* and *Solanum lycopersicum*) at a dose of 0.4 mg/mL. There were no significant effects on *L. perenne* germination and leaf and root growth (14, 34, and 26% inhibition, respectively, after 7 days), a moderate promotion of *L. sativa* root growth (55%), and no effect on *S. lycopersicum* germination (14% inhibition after 7 days) or root growth (Table 1).

Given the strong in vitro nematicidal effects (on J2 mortality and egg hatching) of the extract and its low phytotoxicity on *S. lycopersicum*, an in vivo experiment in pots was conducted. YCC4 extract-treated soil at the LC_50_ concentration (0.44 mg/mL) significantly reduced the reproductive traits of the *M. javanica* population, causing strong reductions (>60%) in nematode egg masses, egg production, and the IF and MR indices with respect to the untreated control after 2 months (Table 2).

**Figure 1:**
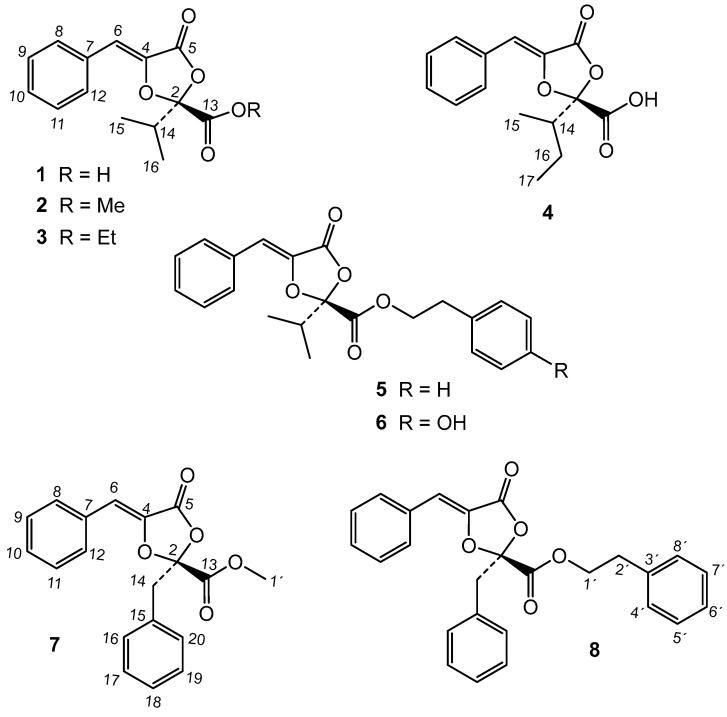
Dioxolanone compounds of *Phyllosticta* sp.

**Figure 2:**
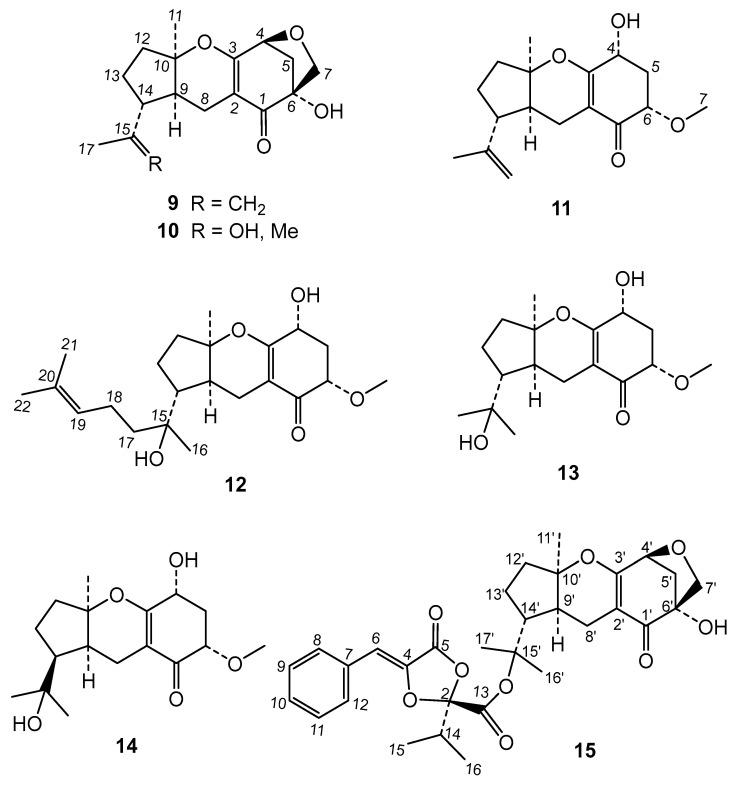
Meroterpene compounds of *Phyllosticta* sp.

The chromatographic study of the extract led to the isolation of two new metabolites and thirteen known compounds (Figure 1 and Figure 2). By comparison of their ^1^H and ^13^C data with the literature, these known compounds were identified as the dioxolanones guignardic acid (**1**) [13,14], ethyl guignardate (**3**) [15], guignardianones A (**5**), C (**2**), D (**8**), and E (**6**) [16,17], phenguignardic acid methyl ester (**7**) [18,19], and the meroterpenes guignardone A (**9**) [20,21], guignardone B (**10**) [20], guignarenone B (**11**), guignarenone C (=guignardone H) (**12**) [22,23], (-)-guignardone I (**13**) [23,24], phyllomeroterpenoid B (**15**) [19], and 3,4-dihydroxybenzoic acid. In this work, we have also completed the assignment of the NMR data of (-)-guignardone I (**13**), whose structure has been recently revised by asymmetric synthesis [22,23].

Among the new compounds, the first one was named metguignardic acid and assigned structure **4**. Its high-resolution mass spectrum showed the molecular ion [M]^+^ at *m*/*z* 276.0999 in accordance with the formula C_15_H_16_O_5_ (Figure S12). The ^1^H NMR spectrum of **4** was very similar to that of guignardic acid (**1**), also obtained from this culture. Analysis of the ^1^H NMR spectrum revealed the presence of two methyl groups at δ 0.99 (t, J = 7.4 Hz, H-17) and 1.07 (d, J = 6.8 Hz, H-15), one methine signal at δ 2.43 (1H, ddd, J = 10.1, 6.8, 3.2 Hz, H-14), and an olefinic proton at δ 6.52 (1H, s). Additionally, the protons of one methylene group were observed at δ 1.29 (1H, ddd, J = 13.5, 10.1, 7.3 Hz, H-16) and 1.66 (1H, ddd, J = 13.5, 7.4, 3.2 Hz-H-16), as well as the resonances of five aromatic protons [7.34 (1H, m), 7.40 (2H, m), 7.67 (2H, dd, J = 7.1, 1.5 Hz)] of a mono-substituted benzene ring. The ^13^C NMR spectrum (Table 3 and Figure S6) combined with the DEPT (Figure S7) and HSQC (Figure S8) spectra confirmed these assignments by showing signals from two methyl groups, a methylene, a methine, and a trisubstituted double bond at δ 110.1 (C-6) and 135.3 (C-4), which is conjugated with a lactone at δ 162.7 (C-5). An angular carbon bearing two oxygenated functions at δ 108.3 (C-2) and a carboxyl group at δ 169.5 (C-13) were also observed. The difference with guignardic acid (**1**) was, therefore, the appearance of a methylene group at δ 22.4 (C-16).

**Figure 3:**
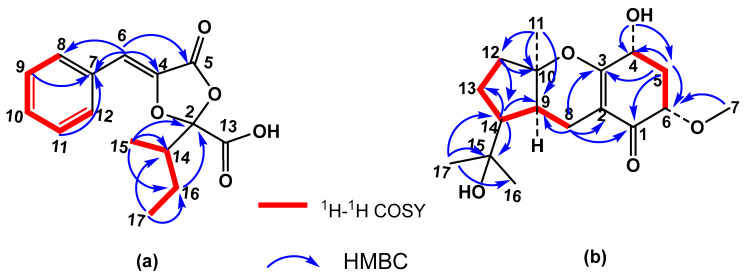
(**a**) Key COSY and HMBC correlations for compound **4**. (**b**) Key COSY and selected HMBC correlations for compound **14**.

The proton and carbon resonances corresponding to the additional methylene H-16, along with those of methyl groups H-15 (δ_C_ 11.0, C-15) and H-17 (δ_C_ 11.5, C-17), and the methine H-14 (δ_C_ 39.3, C-14), were indicative of the presence on C-2 of an isobutyl group instead of an isopropyl one. The planar structure of **4** (Figure 3) was further confirmed by the ^1^H–1H COSY (Figure S9) crosspeaks of H-14–H-15 and H-14–H-16–H-17, and the HMBC (Figure S10) correlations of H-15 and H-17 methyls with C-14 (δ 39.3) and C-16 (δ 22.4). Furthermore, H-15 methyl and H-16 methylene showed connectivities with C-2 (δ 108.3), confirming the position of the isobutyl group in the molecule. The singlet at δ 6.52 (H-6) showed correlations with aromatic carbons at δ 129.9 (C-8 and C-12), 135.3 (C-4), and 162.7 (C-5). Crosspeaks observed between two aromatic protons at δ 7.40 (H-9 and H-11) and a carbon at δ 132.1 permitted this last signal to be assigned to C-7. 

The absolute configuration for **4** was not established. However, the relative configuration of C-2 was considered to be the same as in guignardic acid (**1**) based on the biogenetic relationships of both compounds together with the same negative sign of optical rotation, as published for **1** [13,14], whose absolute configuration has been determined by chemical synthesis [13]. Biogenetically, compound **4** could be derived from the fusion of the products from the oxidative deamination of the amino acids phenylalanine and isoleucine, similarly to that described for guignardic acid (**1**) [13], but replacing the amino acid valine for isoleucine, which would result in the formation of metguignardic acid (**4**) instead of guignardic acid (**1**). There are other dimers of dioxolanone-type compounds in which the amino acids valine or phenylalanine have been proposed as precursors in their formation instead of valine [14,17].

(-)-14-*epi*-Guignardone I is another new natural product to which we have assigned structure **14** on the basis of the following considerations: Its HRESIMS showed the molecular ion [M + Na]^+^ at *m*/*z* 333.1669 corresponding to C_17_H_26_O_5_Na, which indicated that it is an isomeric compound of (-)-guignardone I (**13**) (Figure S23). The ^13^C NMR spectrum (Table 3 and Figure S18) also confirmed the presence of seventeen carbons attributed by the HSQC (Figure S19) experiment to three methyls carbons, one methoxy group, four methylenes, two tertiary carbons at δ 66.0 (C-4) and 79.0 (C-6), two quaternaries carbons bearing oxygen at δ 71.8 (C-15) and 86.3 (C-10), and a carbonyl group at δ 194.6 (C-1). In addition, two olefinic carbons were observed at δ 107.8 (C-2) and 167.5 (C-3), the latter also bound to an oxygen atom.

In its ^1^H NMR spectrum (Figure S17), signals of an angular methyl at δ 1.48 (H-11), two methyls at δ 1.21 and 1.37 (H-16 and H-17) on the same carbon bearing oxygen, and a methoxy group at δ 3.50 (H-7) were observed. Other observed resonances were the signals of coupled protons H-8 at δ 2.04 (1H, ddd, J = 17.0, 10.9, 1.9 Hz, H-8β) and 2.65 (1H, dd, J = 17.0, 6.3 Hz, H-8α) and two doublets at δ 2.18 (J = 13.4, 7.5, 6.0 Hz, H-5β) and 2.40 (J = 13.3, 4.8, 3.8 Hz, H-5α) of the methylene group at C-5. Two methine signals at δ 4.29 (dd, J = 13.2, 5.4 Hz, H-4) and 3.73 (dd, J = 7.9, 3.8 Hz, H-6) were assigned to two geminal protons of hydroxyl and methoxy groups attached at C-4 and C-6 along the signal of proton H-9 at δ 1.88 (dd, J = 10.8, 5.9 Hz, H-9). These assignments were confirmed by COSY, HSQC, and HMBC experiments (Figure 3 and Figures S19–S21). The resonance at δ 2.13 was significant at a lower field than in guignardone I (**13**) (δ 1.59), which was attributed to H-14 based on its HMBC correlations with C-9 (δ 40.8), C-13 (δ 18.9), and C-15 (δ 71.8). This fact led us to think that **14** was the C-14 epimer of (-)-guignardone I, which is rare in this type of meroterpenoid. This relative configuration was finally established considering the NOESY spectrum of **14** (Figure S22), which showed a strong correlation between H-11 methyl with H-9 and H-14, indicating that they have an axial configuration in the α-face of the molecule. Correlations of H-5β with H-4 and H-6 were also observed. These data, along with the coupling constants of the signals corresponding to protons H-4 and H-6, showed an α disposition for the hydroxyl and methoxy group at C-4 and C-6 in the cyclohexenone ring [25].

Compounds **1**–**15** were tested for their biocidal effects against the insect and nematode targets (*S. littoralis*, *M. persicae*, *M. javanica*). Table 4 shows the insect antifeedant effects of these compounds, with **4** and **1** being effective against *S. littoralis* (EC_50_ values of 7.53 and 9.65 μg/cm^2^, respectively), while **1**, **2**, and **4** acted on *M. persicae* (EC_50_ values of 2.87, 1.74, and 11.45 μg/cm^2^, respectively). The positive control thymol was effective against *M. persicae* (EC_50_ value of 7.6 μg/cm^2^) but not on *S. littoralis*.

**Figure 4:**
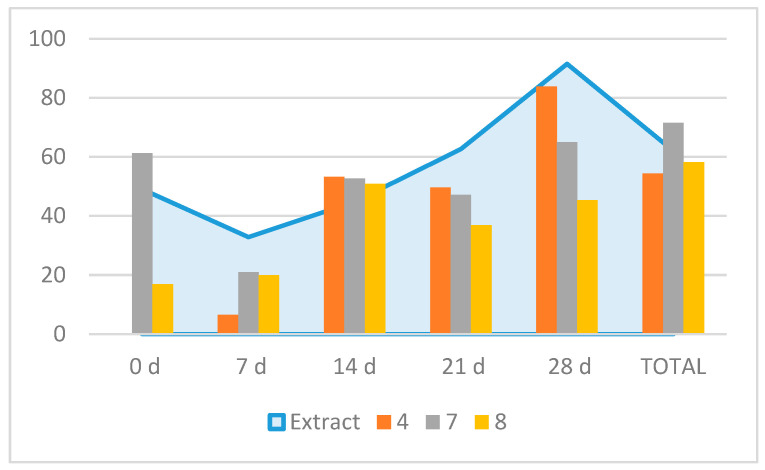
Effects of compounds **4**, **7**, and **8** (extract in the background) on *Meloidogyne javanica* egg hatching. Time 0 d: 5 days of immersion in test solutions; time 7 d and further: number of days of immersion in water after time 0 d. Each column represents the hatch inhibition rate for the respective treatment relative to the control.

When tested against *M. javanica* J2 in vitro, compounds **4**, **7,** and **8** were very active (100% mortality at 1 mg/mL, with LC_50_ values of 0.24, 0.23, and 0.11 mg/mL respectively), with compound **8** being more effective than the positive control thymol (LC_50_ value of 0.13 mg/mL), while **1** showed moderate effects (60% mortality at 1 mg/mL) (Table 5). The time course experiment of the nematicidal compounds (24, 48, and 72 h) showed that **8** was the most active one, starting at 24 h, followed by **4** (Table 5). Further in vitro tests on egg hatching inhibition showed that for the total inhibition rate values, compound **7** was the most active one (71.5% inhibition), followed by **8** (58.2%) and **4** (54.4%) (Figure 4). However, the time course observation showed that **7** induced an early inhibition at 0 d (61.2%) with a similar peak at 28 d (65%); compound **4** peaked at 28 d (83.8%) and compound **8** peaked at 14 d (51%) (Figure 3). Therefore, the time course egg hatching inhibition caused by the extract correlated with the combined action of these compounds.

## 3. Discussion

In this work, the secondary metabolites present in a bioactive extract from the endophytic fungus *Phyllosticta* sp isolated from *P. indica* have been identified. As part of a broad endophytic survey of the Macaronesian laurel forest, *P. capitalensis* (identified as *Guignardia manguiferae*) from *P. indica* was previously described as the producer of a bioactive extract against insects and nematodes [4], but the secondary metabolites responsible for such activities were unknown. 

The chemical study of *Phyllosticta* sp. extract gave the known compounds guignardic acid (**1**), ethyl guignardate (**3**), guignardianones A (**5**), C (**2**), D (**7**), and E (**6**), phenguignardic acid methyl ester (**8**), the meroterpenes guignardone A (**9**) and B (**10**), guignarenone B (**11**) and C (=guignardone H) (**12**), (-)-guignardone I (**13**), and the phyllomeroterpenoid B (**15**), along with the new C-14 epimer of (-)-guignardone I (**14**) and metguignardic acid (**4**). 

*Phyllostica* species and *P. capitalensis* are important sources of meroterpene compounds. The meroterpenes guignardones A-C [20] and guignarerones A-D [22], guignardones D-E and tricycloalternarene F [21], guignarenone C, and guignardone J-K [26] have been isolated from *P. capitalensis* (*G. mangiferae*) isolated from *Ilex cornuta* and guignardones J-O from *Guignardia* sp. [27]. Other compounds from *P. capitalensis* isolated from *Smilax glabra* were 15-hydroxy-tricycloalternarene, guignardones A-B, and guignardones P-S [28]. Guignardones F-I were obtained in cultures of an endophytic fungus A1, isolated from a mangrove plant [23,24]. *P. capitalensis* isolated from *Cephalotaxus fortunei* also yielded a large number of meroterpenes (guignardone B, F, G, C, M, N, O, H, I, J, K; 12-hydroxylated guignardone A, 13-hydroxylated guignardone A, 17-hydroxylated guignardone A, phyllostictone A-E) [15]. A mangrove *P. capitalensis* endophyte gave guignardones A, J, and M and 12-hydroxylated guignardone A [29] and guignardones U-X [30]. Additionally, another isolate of this fungal species produced the sesquiterpene-shikimate-conjugated spirocyclic meroterpenoids A and B [31]. Meroterpenes have been described as antibacterial and anti-inflammatory compounds [31]. However, in this work, we have not found any antifeedant or nematicidal effects for the meroterpenes produced by *P. capitalensis* isolated from *P. indica.*

In contrast to meroterpenes, a lower number of dioxolanone derivatives have been found in *Phyllostica* species. An isolate of *P. capitalensis* from *Smilax glabra* gave guignardiones C-D [32], and an isolate from *Cephalotaxus fortunei* yielded the dioxolanones guignardic acid, 2-hydroxyethyl guignardate, ethyl guignardate, and guignardianone C [15]. Guignardianone G was reported from a mangrove isolate of *P. capitalensis* [33]. Plant protection effects for dioxolanone-type compounds have been described. Guignardianone C showed broad-spectrum antifungal activities against the plant pathogens *Rhizoctonia solani*, *Fusarium graminearum*, and *Botrytis cinerea*, but not guignardic or penguignardic acids [30]. Guignardic and alaguignardic acids were phytotoxic in a *Vitis vinifera* leaf-disk assay and also against *Oryza sativa* and *Triticum aestivum*, whereas guignardianones A-F were not active, indicating that the free carboxyl group or at least a polar moiety is important for the phytotoxic activity of the analyzed dioxolanone-type secondary metabolites [17]. Guignardianone C moderately inhibited the growth of *L. sativa* and *L. perenne* [15].

In this work, only dioxolanone derivatives were active. Guignardic (**1**) and metguignardic acid (**4**) were effective antifeedants against both insect species, while guignardione C (**2**) was only active on the aphid *M. persicae*. In this case, and similarly to the phytotoxic activity described before, the active compounds presented a free carboxyl group (**1**, **4**) or a metyhylated group (**2**) in contrast with the inactive less polar ethyl derivative one (**3**). The nematicidal effects showed a different structure–activity relationship pattern. The presence of an aromatic phenylethyl substituent in C-2 determined a strong effect (**7, 8**), followed by an isobutyl group (**4**). This is the first report on the insect antifeedant or nematicidal effects of these compounds.

## 4. Materials and Methods

### 4.1. General Experimental Procedures 

Optical rotations were determined at room temperature on a Perkin Elmer 343 polarimeter (Perkin Elmer, Waltham, MA, USA). IR spectra were taken in Bruker (Billerica, MA, USA) IFS 66/S and Perkin-Elmer 1600 spectrometers (Perkin Elmer, Waltham, MA, USA). NMR spectra were measured on a Bruker AMX-500 spectrometer (^1^H 500 MHz/^13^C 125 MHz, Bruker, Billerica, MA, USA) with a pulsed field gradient using the CDCl_3_ solvent (δ_H_ 7.26 and δ_C_ 77.0) as the internal standard. EIMS and exact mass measurements were recorded on a Micromass Autospec (Manchester, UK) instrument at 70 eV. The HRESIMS were acquired using a Micromass LCT Premier (Waters, Manchester, UK) spectrometer. Preparative and semipreparative HPLC was carried out with a Beckman Coulter 125P equipped with a diode-array detector 168 (Beckman Coulter Life Sciences, Brea, CA, USA) using preparative Interstil Prep-sil 20 mm × 250 mm, 10 µm particle size (Gasukuro Kogyo, Tokyo, Japan) and semipreparative Beckman (Brea, CA, USA) Ultrasphere silica 10 mm × 250 mm, 5 µm particle size columns. Silica gel 60 F_254_ (Merck, Darmstadt, Germany) and Sephadex LH-20 (Sigma-Aldrich, St. Louis, MO, USA) were used for column chromatography.

### 4.2. Isolation and Identification of Endophytic Fungus YCC4

The endophytic fungus YCC4 was isolated from leaves of the endemic Macaronesian species *Persea indica* (L.) Spreng., collected at Las Mercedes in Parque Rural de Anaga (Tenerife, Canary Islands). The leaves were washed with tap water for 10 min to remove impurities. Surface disinfection was performed in a laminar flow hood by soaking in 70% ethanol for 1 min, followed by 1% sodium hypochlorite for 10 min, and then washed again with 70% ethanol for 1 min. The plant samples were finally washed with sterile distilled water for 1 min. Surface-disinfected samples were then cut into small segments (0.5 cm) and tissue segments were placed in Petri dishes containing sterile potato dextrose agar medium (PDA), supplemented with chloramphenicol (50 mg/L) to inhibit bacterial growth. The plates were incubated at 27 °C in a growth chamber for 3–15 days in darkness. The emerging fungal colonies were transferred to fresh PDA plates to obtain pure strain and further identification.

The isolated fungus was identified at the molecular level based on the amplification (PCR) and sequencing of the ribosomal ITS region of the rDNA, according to a molecular biological protocol described previously [34]. Briefly, genomic DNA (100–200 ng) was amplified (PTC-200 Thermal Cycler, MJ Research, San Diego, CA, USA), 25 µL final volume, with the AmpONE Taq DNA polymerase PCR kit (GeneAll, Seoul, Republic of Korea) with 35 cycles (95 °C, 1 min; 50 °C, 20 s; 72 °C, 1.5 min) after an initial denaturation (95 °C, 2 min) followed by a final extension (72 °C, 7 min). The PCR-amplified products were checked by agarose gel (1%) electrophoresis, purified using the EXO-SAP-IT kit (Affimetrix-USB; Thermo Fisher Scientific, Waltham, MA, USA), and sequenced on an AB 3500 Genetic Analyzer (Thermo Fisher Scientific, Waltham, MA, USA) at the University of La Laguna (La Laguna, Spain) genomic service. The obtained ITS1-5.8S-ITS2 sequence data (Sequence data in Supplementary Materials) were compared with those published in the NCBI (National Center for Biotechnology Information, https://www.ncbi.nlm.nih.gov/ accessed on 10 March 2024) database using the Basic Local Alignment Search Tool (nBLAST 2.13.0). A total of fifty sequences were selected from Genbank to conduct maximum likelihood phylogenetic analysis using MEGA version 11 (Mega Limited, Auckland, New Zealand) under the general time reversible and gamma distribution (GTR+G) parameter model. As result, the endophytic strain YCC-4 was determined as *Phyllostica* sp. (MH393361). A sample of this *Phyllosticta* isolate was deposited in CECT (Valencia, Spain) with number 20,914 in accordance with the Budapest treaty.

### 4.3. Cultivation of YCC4 for Extract Preparation

*Phyllosticta* sp. was cultivated in PDA medium in Petri dishes for 15 days at 26 °C. After this time, sterile distilled water (10 mL) was added, and the surface of the mycelium was gently scraped with a spatula. This mycelial suspension was poured into an Erlenmeyer flask (250 mL) with 100 mL of Czapek-Dox-Yeast liquid medium [Cz-L: NaNO_3_ (2 g/L), KH_2_PO_4_ (5 g/L), MgSO_4_ (0.5 g/L), FeSO_4_ (0.01 g/L), ZnSO_4_ (0.003 g/L), yeast extract (1 g/L) and glucose (60 g/L)] and cultivated at 26 °C by continuously stirring (120 rpm) for 4 days to obtain 1 L of pre-inoculum. Erlenmeyer flasks (100, 250 mL) with fresh medium (100 mL) were inoculated with 5 mL of the pre-inoculum. Then, after 20 days of fermentation under the same conditions, the culture medium was separated from the mycelium by filtration in a Büchner and extracted with EtOAc. Subsequently, the solvent was removed under reduced pressure to afford a dry crude extract (10.5 g).

### 4.4. Isolation and Compound Identification 

The dry crude extract (10.5 g) was fractionated by vacuum liquid chromatography (VLC) over silica gel eluted with an increasingly polar gradient of n-Hexane/EtOAc and EtOAc/MeOH to obtain six fractions. Fraction 1 (n-Hexane/EtOAc 75:25) was separated into four subfractions (F1A-F1D) by silica gel column chromatography using n-hexane/EtOAc mixtures of increasing polarity (90:10–50:50). Ethyl guignardate (**3**) (28 mg, t_R_ = 14 min), guignardianone A (**5**) (23.0 mg, t_R_ = 27 min), and guignardianone C (**2**) (96.1 mg, t_R_ = 31 min) were isolated from the less polar subfraction 1A (n-hexane/EtOAc, 90:10) using preparative high-pressure liquid chromatography (HPLC) (Hex/EtOAc 97:3). Subfraction 1B, obtained with n-hexane/EtOAc 15%, was subjected to (CC) on a silica gel medium-pressure column with n-hexane/EtOAc gradients (100:0–90:10) and, subsequently, by preparative HPLC using n-hexane/EtOAc (97:3) as the mobile phase afforded guignardianone D (**8**) (89 mg, t_R_ = 23 min) and penguignardic acid methyl ester (**7**) (36.5 m, t_R_ = 36 min). Similarly, the preparative HPLC of subfraction 1C (n-hexane/EtOAc 85:15) eluted with n-hexane/EtOAc 20% gave guignardone A (**9**) (78.3 mg, t_R_ = 20 min). In the most polar subfraction of the chromatography of fraction 1 (n-hexane/EtOAc 50:50), metguignardic acid (**4**) (204 mg) was isolated. 

Guignardianone A (**5**) (430 mg) and guignardone A (**9**) were obtained again (80 mg) from fraction 2 by silica gel column chromatography with mixtures of increasing polarity of n-hexane/EtOAc and EtOAc/MeOH. In addition, guignardone B (**10**) (13.6 mg) and phyllomeroterpenoid B (**15**) (5.0 mg) were isolated in the fractions eluted with EtOAc. Further purification of the most polar fractions (MeOH) by HPLC with n-hexane/EtOAc/MeOH (30:64:6) as the mobile phase gave metguignardic acid (**4**) (30 mg, t_R_ = 27 min) and guignardic acid (**1**) (118 mg, t_R_ = 34 min).

Fraction 3, obtained by eluting with n-hexane/EtOAc (50:50), was chromatographed on a silica gel column with mixtures of increasing polarity of n-hexane/EtOAc and EtOAc/MeOH. After successive medium-pressure column chromatographies and/or HPLC, guignardianone C (**2**) (49 mg), phenguignardic acid methyl ester (**8**) (2.4 mg), guignardianone E (**6**) (1.5 mg), guignarenone C [=guignardone H] (**12**) (14.6 mg), guignardone B (**10**) (19.2 mg), and 3,4-dihydroxybenzoic acid (34.3 mg) were isolated. 

The most polar fractions 4–6 (EtOAc/MeOH 100:0–50:50) of the extract were subjected to silica gel column chromatography eluted with an increasingly polar gradient of CH_2_Cl_2_/MeOH (99:2–0:100). Less polar fractions (CH_2_Cl_2_/MeOH, 99:1–99:2) were purified by semipreparative HPLC using a mixture of CH_2_Cl_2_/EtOAc 50% to give guignarenone B (**11**) (2.2 mg, t_R_ = 17 min). Fractions eluted with CH_2_Cl_2_/MeOH (99:5–90:20) were isolated (-)-14-*epi*-guignardone I (**14**) (1.1 mg, t_R_ = 25 min) and (-)-guignardone I (**13**) (6.2 mg, t_R_ = 27 min) by HPLC with n-hexane/EtOAc/MeOH (40:55:5) as the eluent. 

#### 4.4.1. Guignardic Acid (**1**)

[α]_D_: −26.7 (c 0.25, CHCl_3_); ^1^H NMR (500 MHz, CDCl_3_): δ 1.10 and 1.09 (each 3H, d, J = 6.9 Hz, H-15 and H-16), 2.68 (1H, sept, J = 6.9 Hz, H-14), 6.53 (1H, s, H-6), 7.35 (1H, m, H-10), 7.39 (2H, m, H-9 and H-11), 7.68 (2H, br d, J = 7.1 Hz, H-8 and H-12); EIMS *m*/*z*: 262 [M]^+^ (5), 118 (100), 90 (38); ^13^C NMR data, see Table 3; HREIMS [M]^+^ at *m*/*z* 262.0853, calculated for C_14_H_14_O_5_ 262.0841.

#### 4.4.2. Guignardianone C (**2**)

[α]_D_: −10.2 (c 0.25, CHCl_3_); ^1^H-RMN (500 MHz, CDCl_3_): δ 1.07 and 1.08 (6H, d, J = 6.9 Hz, H-15 and H-16), 2.69 (1H, sept, J = 6.9 Hz, H-14), 3.85 (3H, s, -OCH_3_), 6.50 (1H, s, H-6), 7.35 (1H, m, H-10), 7.41 (2H, m, H-9 and H-11), 7.68 (2H, d, J = 7.1 Hz, H-8 and H-12); EIMS *m*/*z*: 276 [M]^+^ (5), 118 (100), 90 (39), 71 (13); ^13^C NMR data, see Table 3; HREIMS [M]^+^ at *m*/*z* 276.0999, calculated for C_15_H_16_O_5_ 276.0998.

#### 4.4.3. Metguignardic Acid (**4**) 

[α]_D_: −54.7 (c 0.3, CHCl_3_); IR (CHCl_3_) ν_max_: 2919, 1796, 1739, 1254, 1195, 1183 cm^−1^; ^1^H NMR (500 MHz, CDCl_3_): δ 0.99 (3H, t, J = 7.4 Hz, H-17), 1.07 (3H, d, J = 6.8 Hz, H-15), 1.29 (1H, ddd, J = 13.5, 10.1, 7.3 Hz, H-16), 1.66 (1H, ddd, J = 13.5, 7.4, 3.2 Hz, H-16), 2.43 (1H, ddd, J = 10.1, 6.8, 3.2 Hz, H-14), 6.52 (1H, s, H-6), 7.34 (1H, m, H-10), 7.40 (2H, m, H-9 and H-11), 7.67 (2H, dd, J = 7.1, 1.5 Hz, H-8 and H-12); EIMS: *m*/*z* 276 [M]^+^ (6), 118 (100), 90 (50); ^13^C NMR data, see Table 3; HREIMS [M]^+^ at *m*/*z* 276.0999, calculated for C_15_H_16_O_5_ 276.0998.

#### 4.4.4. Phenguignardic Acid Methyl Ester (**7**)

[α]_D_ +47.6 (c 0.14, CHCl_3_); IR (CHCl_3_) ν_max_: 2919, 2369, 2345, 1734, 1256, 1178 cm^−1^; ^1^H NMR (500 MHz, CDCl_3_): δ 3.52 and 3.57 (each 1H, d, J = 14.7 Hz, H-14), 3.85 (3H, s, -OCH_3_), 6.30 (1H, s, H-6), 7.24 (3H, m, H-17, H-18 and H-19), 7.28 (2H, m, H-16 and H-20), 7.36 (1H, m, H-10), 7.40 (2H, m, H-9 and H-11), 7.63 (2H, br d, J = 7.2 Hz, H-8 and H-12); ^13^C NMR data, see Table 3; EIMS: *m*/*z* 324 [M]^+^ (2), 264 (2), 118 (100), 91 (29); HREIMS [M]^+^ at *m*/*z* 324.0987, calculated for C_19_H_16_O_5_ 324.0998.

#### 4.4.5. Guignardianone D (**8**)

[α]_D_: +40.3 (c 0.25, CHCl_3_); ^1^H-RMN (500 MHz, CDCl_3_): δ 2.96 (2H, t, J = 6.6 Hz, H-2′), 3.52 and 3.46 (each 1H, d, J = 14.7 Hz, H-14), 4.45 (2H, m, H-1′), 6.30 (1H, s, H-6), 7.15 (2H, m, H-4′ and H-8′), 7.19 (3H, m, H-5′, H-6′and H-7′), 7.24 (5H, br s, H-16, H-17, H-18, H-19 and H-20), 7.36 (1H, m, H-10), 7.41 (2H, m, H-9 and H-11), 7.62 (2H, d, J = 7.2 Hz, H-8 and H-12); EIMS: *m*/*z* 414 [M]^+^ (4), 265 (11), 118 (100), 105 (27), 91 (84); ^13^C NMR data, see Table 3; HREIMS [M]^+^ at *m*/*z* 414.1465, calculated for C_26_H_22_O_5_ 414.1467.

#### 4.4.6. Guignarenone C (=Guignardone H) (**11**)

[α]_D_: +82.8 (c 0.18, CHCl_3_); ^1^H NMR (500 MHz, CDCl_3_): δ 1.33 (3H, s, H-11), 1.54 (1H, dddd, J = 13.2, 11.1, 8.4, 3.9 Hz, H-13α), 1.66 (3H, br s, H-17), 1.79 (1H, ddd, J = 14.1, 11.2, 7.1 Hz, H-12α), 1.92 (1H, m, H-13β), 1.95 (1H, m, H-9), 2.13 (1H, m, H-12β), 2.17 (1H, m, H-8α), 2.20 (1H, m, H-14), 2.23 (1H, ddd, 13.8, 6.7, 5.3 Hz, H-5α), 2.33 (1H, dt, J = 16.2, 1.5 Hz, H-8β), 2.37 (1H, ddd, J = 13.8, 4.8, 3.7 Hz, H-5β), 3.47 (3H, s, -OMe), 3.70 (1H, dd, J = 6.7, 3.6 Hz, H-6), 4.25 (1H, t, J = 5.1 Hz, H-4), 4.63 (1H, dt, J = 1.8, 0.9 Hz, H-16), 4.73 (1H, t, J = 1.5 Hz, H-16); ^13^C NMR data, see Table 1; EIMS *m*/*z*: 292 [M]^+^ (1), 262 (100), 234 (18), 219 (10), 122 (32), 84 (42); ^13^C NMR data, see Table 3; HREIMS [M]^+^ at *m*/*z* 292.1676, calculated for C_17_H_24_O_4_ 292.1675.

#### 4.4.7. (-)-Guignardone I (**13**)

[α]_D_: −40.0 (c 0.3, MeOH); ^1^H NMR (500 MHz, CDCl_3_): δ 1.19 and 1.21 (each 3H, s, H-16 and 17), 1.34 (3H, s, H-11), 1.57 and 1.80 (each 1H, m, H-13β and H-13α), 1.59 (1H, m, H-14), 1.63 and 2.02 (each 1H, m, H-12β and H-12α), 2.06 (1H, td, J = 9.0, 1.5 Hz, H-9), 2.23 (1H, ddd, J = 13.7, 7.0, 5.5, H-5β), 2.26 (1H, ddd, J = 17.7, 7.5, 1.5 Hz, H-8β), 2.39 (1H, ddd, J = 13.7, 4.8, 3.7 Hz, H-5α), 2.64 (1H, d, J = 17.5 Hz, H-8α), 3.48 (3H, s, -OCH_3_), 3.72 (1H, dd, J = 6.8, 3.7 Hz, H-6), 4.27 (1H, br t, J = 5.1 Hz, H-4); EIMS: *m*/*z* 310 [M]^+^ (4), 280 (45), 262 (22), 209 (21), 194 (100), 179 (25), 166 (37), 122 (90), 81 (49), 59 (58); ^13^C NMR data, see Table 3; HREIMS [M]^+^ at *m*/*z* 310.1769, calculated for C_17_H_26_O_5_ 310.1780.

#### 4.4.8. (-)-14-*epi*-Guignardone I (**14**)

[α]_D_: +6.25 (c 0.32, MeOH); ν_max_: 3448, 2969, 2929, 1654,1617, 1457, 1362, 1283, 1215, 1163, 1119, 1071 cm^−1^; ^1^H NMR (500 MHz, CDCl_3_): δ 1.21 and 1.37 (each 3H, s, H-16 and 17), 1.48 (3H, s, H-11), 1.69 and 1.94 (each 1H, m, H-13), 1.82 (2H, br t, J = 8 Hz, H-12), 1.88 (1H, dd, J = 10.8, 5.9 Hz, H-9), 2.04 (1H, ddd, J =17.0, 10.9, 1.9 Hz, H-8β), 2.13 (1H, m, H-14), 2.18 (1H, m, H-5β), 2.40 (1H, ddd, J = 13.3, 4.8, 3.8 Hz, H-5α), 2.65 (1H, dd, J = 17.0, 6.3 Hz, H-8α), 3.18 (d, J = 6.3, -OH), 3.50 (3H, s, -OCH_3_), 3.73 (1H, dd, J = 7.9, 3.8 Hz, H-6), 4.29 (1H, dt, J = 6.4, 4.6, H-4); ^13^C NMR data, see Table 3; HRESIMS [M+Na]^+^ at *m*/*z* 333.1669, calculated for C_17_H_26_O_5_Na 333.1678.

### 4.5. Nematicidal Activity

The *Meloidogyne javanica* population was maintained on *Solanum lycopersicum* (var. Marmande) plants cultivated in pot cultures in environmentally controlled growth chambers (at 25 ± 1 °C, >70% relative humidity). Egg masses of *M. javanica* were handpicked from infected tomato roots. Second-stage juveniles (J2) were obtained from hatched eggs by incubating egg masses in a water suspension at 25 °C for 24 h. 

#### 4.5.1. In Vitro Effect on Juveniles

The extract, fractions, and compounds were dissolved in distilled water containing 5% of a DMSO-Tween solution (0.5% Tween 20 in DMSO) and evaluated as described by Andres et al. (2017) [35]. The initial concentrations tested were of 1 and 0.5 mg mL for extract or pure compound, respectively, and four replicates were used for each test. Tests with mortality rates >90% at 72 h were further tested to assess J2 mortality after 24 and 48 h. The nematicidal activity data are presented as percent dead J2 corrected according to Scheider–Orelli’s formula. Five serial dilutions were used to calculate the effective lethal doses (LC_50_ and LC_90_) by Probit Analysis (STATGRAPHICS Centurion XVI, version 16.1.02).

#### 4.5.2. In Vitro Effect on Egg Hatching

Egg masses (three) were placed in each well of a 96-well plate containing test solutions at LC_90_ concentrations. The control wells contained water/DMSO/Tween 20. Each experiment was replicated four times. The plates were covered and maintained in the dark at 25C for 5 days, after which the hatched J2s were counted, and the treatments were replaced with sterilized distilled water. The hatched J2s from egg masses were monitored weekly for 1 month until egg hatching was finished in the control [35]. Relative hatch inhibition rates (compared with the controls) were calculated for each immersion time as follows:

Relative suppression rate (%) = (number of J2 in control − number of J2 in test solutions)/number of J2 in control × 100.

#### 4.5.3. Effect on Infection and Reproduction of *M. javanica* Population in Tomato Plants Hatching

The extract was evaluated at 0.45 mg/mL (LC_50_) in 1% ethanol. The treatment (100 mL) was applied to a pot containing 1000 g of the moistened substrate (sterile sandy/loam soil mixture) at the time of nematode inoculation (2000 *M. javanica* eggs) and incubated for 5 days in a growth chamber (25 °C, 60% relative humidity). After this period, 4-week-old tomato seedlings were transplanted, maintained for 60 days in a growth chamber (25 °C, 60% relative humidity, 16 h photoperiod), and fertilized with 50 mL of a 0.3% solution of 20-20-20 (N-P-K) every 10 days. Six pots for each treatment were used, and the experiment was carried out in duplicate. At harvest, the whole root system from each pot was collected. Roots were washed free of soil, examined for determining the number of egg masses. Eggs from root egg masses were extracted by maceration in a 10% commercial bleach solution (40 g/L NaOCl) for 10 min, passed through a 70 μm aperture screen, and collected in a 25 μm sieve for final counting. The relative suppression rate of the extract on egg masses and number of eggs per plant was calculated. The infection frequency (IF: number of egg masses per plant divided by the number of eggs inoculated per pot) and the multiplication rate (MR: number of eggs per plant divided by the egg inoculum) were determined. Data from each treatment were transformed by Log10 (x); mean values were compared by Student’s *t*-test at *p* < 0.05 to determine significant differences in the nematode population’s reproductive traits associated with treatment effects.

### 4.6. Antifeedant Activity

The insect colonies (*Spodoptera littoralis*, *Myzus persicae* and *Rhopalosiphum padi*) come from laboratory colonies reared on artificial diet and host plants (*Capsicum annuum*, *Hordeum vulgare*), respectively, at 22 ± 1 °C, >70% relative humidity and 16:8 h (L:D) photoperiod at ICA-CSIC.

The tests were described before [36]. Briefly, the upper surface of leaf disks or fragments (1.0 cm^2^) of *C. annuum* and *H. vulgare* were treated with 10 µL of extract or compound at an initial dose of 10 or 5 µg/µL (100 or 50 µg/cm^2^), respectively. Two sixth-instar *S. littoralis* larvae (>24 h after molting) per Petri dish or 10 apterous aphid adults (24–48 h old) placed in a 2 × 2 cm ventilated plastic box (20) were allowed to feed at room temperature or in the growth chamber. The experiments ended at 75% larval consumption of the paired control or treatment disks for *S. littoralis* or after 24 h for aphids. Each experiment was repeated 2 times. Feeding inhibition (%FI), based on the disk surface consumption (digitalized with https://imagej.nih.gov/ij/ accessed on 27 November 2023) [37], and aphid settling inhibition (%SI), based on the number of aphids on each leaf fragment, were calculated as % FI/SI = [1 − (T/C) × 100], where T and C represent feeding/settling on treated and control leaf disks, respectively. The significance of these effects was analyzed by the nonparametric Wilcoxon paired signed-rank test. Tests with an FI/SI > 70% were further tested in dose–response experiments (range of activities between 100 and <50%, minimum of 3 doses) to calculate their effective dose EC_50_ from linear regression analysis (% FI/SI on Log-dose, STATGRAPHICS Centurion XVI, version 16.1.02).

## 5. Conclusions

The endophyte *Phyllosticta* sp. has been isolated from *Persea indica*. An EtOAc extract from this fungus showed strong insect antifeedant (against *Spodoptera littoralis* and *Myzus persicae*) and nematicidal (against *Meloydogine javanica*) effects in vitro and in vivo.

A comprehensive chemical study of this extract gave the known compounds guignardic acid (**1**), ethyl guignardate (**3**), guignardianones A (**5**), C (**2**), D (**7**), and E (**6**), phenguignardic acid methyl ester (**8**), the meroterpenes guignardone A (**9**) and B (**10**), guignarenone B (**11**) and C (=guignardone H) (**12**), (-)-guignardone I (**13**), and the phyllomeroterpenoid B (**15**), along with the new C-14 epimer of (-)-guignardone I (**14**) and metguignardic acid (**4**). Among these compounds, only dioxolanone derivatives were active against the insect and nematode targets. Compounds **1** and **4** were effective antifeedants against both insect species, while **2** was only active on the aphid *M. persicae*. The nematicidal compounds were **4, 7**, and **8**. This is the first report on the insect antifeedant or nematicidal effects of these compounds. Since the insect antifeedant and nematicidal activity of the *Phyllosticta* sp. extract depends on the presence of dioxolanone components, future fermentation optimizations are needed to promote the biosynthesis of these compounds instead of the meroterpenes.

## 6. Patents 

Gonzalez-Coloma, A.; Díaz, C.E.; Andres, M.F.; Fraga, B.M.; Bolaños, P. et al. Biocidal products and use thereof for controlling phytopathogens and pest organisms that harm plants. **2016**, PCT Patent WO 2016/034751 A1 

## Figures and Tables

**Table 1:** Summary of the bioactivities of the YCC4 extract on the insects (*Spodoptera littoralis*, *Myzus persicae*, *Rhopalosiphum padi*), the nematode *Meloydogine javanica*, and the plant models *Lolium perenne*, *Lactuca sativa*, and *Solanum lycopersicum*.

Target	Action	Value
*S. littoralis*	Antifeedant	9.77 (4.13–16.95) ^a^
*M. persicae*	Antifeedant	17.2 (4.13–23.16) ^a^
*R. padi*	Antifeedant	>100
*M. javanica*	J2 mortality	0.44 (0.41–0.47) ^b^
	Egg hatching (28 days)	64 ^c^
*L. perenne*	Germination	85.7 ± 21.4 ^d^
	Root growth	74.4 ± 7.1 ^d^
	Leaf growth	65.8 ± 7.8 ^d^
*L. sativa*	Germination	100 ^d^
	Root growth	155.3 ± 22.9 ^d^
*S. lycopersicum*	Germination	85.7 ± 21.4 ^d^
	Root growth	107.7 ± 13.1 ^d^

^a^ Effective dose EC_50_ (µg/cm^2^). ^b^ Lethal concentration LC_50_ (mg/mL). ^c^ Percent inhibition. ^d^ Percent relative to the control.

**Table 2:** In vivo effects of the *Phyllosticta capitalensis* extract on reproductive traits of *Meloidogyne javanica* in tomato plants, 60 days post-inoculation, with 2000 eggs per plant, maintained in a growth chamber.

Treatment	Egg Masses/Plant ^a^	RS ^b^ %	Eggs/Plant ×100	RS ^c^ %	IF ^d^	MR ^e^
Extract	47.4 ± 6.2 ^a^	66.6	335 ± 5.7 ^a^	60	0.0237 ^a^	16.6 ^a^
Control	142 ± 22.5 ^b^		832 ± 5.5 ^b^		0.71 ^b^	41.2 ^b^

^a^ Values are mean ± standard error of ten replicated plants. Values within the same column followed by different lower-case letters are significantly different according to least significance. ^b^ Relative suppression of eggs masses difference (LSD) test (*p* < 0.05). ^c^ Relative suppression of number of eggs per plant. ^d^ Infection frequency: egg masses per plant/egg inoculum. ^e^ Multiplication rate: eggs per plant/egg inoculum.

**Table 3:** ^13^C NMR data of compounds **1**–**4**, **7**–**8**, and **11**–**14**.

Carbon	1	2	4	7	8	11	13	14
1						195.0	194.9	194.6
2	109.6	108.5	108.3	105.2	105.2	105.9	105.5	107.8
3						167.7	168.4	167.5
4	136.0	135.7	135.3	135.3	135.2	65.7	65.8	66.0
5	163.5	162.8	162.7	162.3	162.3	34.5	34.6	34.8
6	109.4	109.6	110.1	109.5	109.6	79.1	79.1	79.0
7	132.2	132.3	132.1	132.2	132.2	58.4	58.4	58.5
8	129.9	129.9	129.9	129.8	129.8	16.1	18.7	16.1
9	128.7	128.8	128.8	128.8	128.7	43.2	41.3	40.8
10	129.0	129.1	129.2	129.8	129.0	87.7	88.9	86.3
11	128.7	128.8	128.8	128.8	128.7	22.3	22.2	30.5
12	129.9	129.9	129.9	129.0	129.8	37.4	38.4	34.7
13	169.5	166.0	169.5	165.2	165.8	26.9	24.6	18.9
14	33.1	33.0	39.3	40.7	40.8	48.9	51.1	49.6
15	14.4	14.5	11.1	130.6	130.6	145.5	73.0	71.8
16	15.2	15.2	22.4	131.0	131.0	111.2	27.7 ^a^	27.4 ^b^
17			11.5	128.6	128.5	19.2	28.7 ^a^	29.5 ^b^
18				127.8	127.9			
19				128.6	128.5			
20				131.0	131.0			
1′		53.4		67.4	53.6			
2′				34.8				
3′				136.8				
4′/8′				128.7				
5′/7′				128.4				
6′				126.8				

^a,b^ These carbons can be interchanged.

**Table 4:** Insect antifeedant effects of compounds **1**–**5**, **7**–**12**, and **14**–**15** tested at 50 µg/cm^2^ (data are expressed as average ± standard error) and effective doses EC_50_.

Chemical Class	Compound	*S. littoralis* %FI ± SE	EC_50_ (µg/cm^2^)	*M. persicae* %SI ± SE	EC_50_ (µg/cm^2^)
Dioxolanone	**1**	84.4 ± 4.9	9.6 (5.3–17.5)	82.0 ± 5.0	2.9 (1.0–7.9)
	**2**	50.3 ± 17.9		82.9 ± 3.7	1.7 (0.9–3.4)
	**3**	40.8 ± 15.3		29.1 ± 8.0	
	**4**	94.5 ± 1.94	7.5 (5.4–10.4)	86.6 ± 3.5	11.4 (8.3–15.8)
	**5**	52.0 ± 13.6		31.5 ± 8.3	
	**7**	20.0 ± 8.8		64.5 ± 7.9	
	**8**	28.3 ± 14.1		50.7 ± 7.7	
Meroterpene	**9**	41.3 ± 16.1		37.3 ± 7.9	
	**10**	7.3 ± 7.3		47.0 ± 7.3	
	**11**	60.5 ± 16.9		44.1 ± 7.3	
	**12**	56.7 ± 14.0		35.2 ± 8.3	
	**14**	53.7 ± 12.5		39.5 ± 8.0	
Phyllomeroterpenoid	**15**	55.2 ± 16.3		52.2 ± 8.5	

**Table 5:** Nematicidal activity against *Meloidogyne javanica* J2 of compounds **1**–**5**, **7**–**12**, and **14**–**15**. Data are expressed as average ± standard error and lethal doses (LC_50_ and LC_90_).

Compound ^a^	*Meloidogyne javanica* (%) ^b^				
	24 h ^c^	48 h ^c^	72 h ^c^	LC_50_ mg/mL ^d^ (95% CLs)	LC_90_ mg/mL ^d^ (95% CLs)
**1**			60.98 ± 3.42		
**2**			12.11 ± 0.32		
**3**			8.60 ± 2.81		
**4**	86.68 ± 0.95	88.30 ± 2.51	100.0 ± 0.0	0.24 (0.23–0.25)	0.40 (0.39–0.43)
**5**			5.78 ± 1.37		
**7**	40.98 ± 2.54	97.13 ± 0.51	100.0 ± 0.0	0.23 (0.22–0.24)	0.43 (0.41–0.45)
**8**	94.23 ± 3.59	100 ± 0.00	100.0 ± 0.0	0.11 (0.10–0.11)	0.19 (0.18–0.20)
**9**			49.09 ± 0.78		
**10**			0.00 ± 0.18		
**11**			0.00 ± 0.63		
**12**			0.05 ± 0.37		
**14**			0.00 ± 0.29		
**15**			7.45 ± 1.79		

^a^ Values (%) are means of four replicates. ^b^ Tested at 0.5 mg/mL. ^c^ Incubation time. ^d^ Lethal doses (LC_50_ and LC_90_) and 95% confidence limits (CLs).

## Data Availability

Data are contained within the article and Supplementary Materials.

## Supplementary Materials

The following supporting information can be downloaded at: https://www.mdpi.com/article/10.3390/molecules29194568/s1, Data sequence of *Phyllosticta* sp.; Figures S1–S4: ^1^H-NMR and ^13^C-NMR of compounds **1**–**2**; Figures S5–S12: ^1^H-NMR,^13^C-NMR, DEPT, HSQC, COSY, HMBC, EIMS, and HREIMS spectra for compound **4**; Figures S13–S16: ^1^H-NMR and ^13^C-NMR of compounds **7**–**8**; Figures S17–S23: ^1^H-NMR,^13^C-NMR, HSQC, COSY, HMBC, NOESY, and HRESIMS spectra for compound **14**.
