# Supplementary material for: Nematicidal and Insecticidal Compounds from the Laurel Forest Endophytic Fungus Phyllosticta sp
Source: Molecules. 2024 Sep 26;29(19):4568. doi: 10.3390/molecules29194568 (PMC11477566; doi:10.3390/molecules29194568)
Supplement: Supplementary file 1 [file molecules-29-04568-s001.zip › molecules-3156205-supplementary.pdf]

Nematicidal and insecticidal compounds from the Laurel Forest endophytic fungus *Phyllosticta sp*.

Carmen E. Díaz*[,1], María Fe Andrés[2], Patricia Bolaños[1], Azucena González-Coloma*[, 2]

[†] *Instituto de Productos Naturales y Agrobiología, C.S.I.C., Avda. Astrofísico F. Sánchez 3, 38206-La Laguna, Tenerife, Canary Islands, Spain*

[‡] *Instituto de Ciencias Agrarias, CSIC, Serrano 115-dpdo, 28006 Madrid, Spain*

*Corresponding authors:

E-mail address: celisa@ipna.csic.es (C.E. Díaz)

E-mail address: azu@ica.csic.es (A. González-Coloma)

List of contents:

Sequence data of endophytic fungus YCC4

Figure S1. ^{1}H-NMR spectrum of compound **1** ($CDCl_3$, 500 MHz)

Figure S2. ^{13}C-NMR spectrum of compound **1** ($CDCl_3$, 125 MHz)

Figure S3. ^{1}H-NMR spectrum of compound **2** ($CDCl_3$, 500 MHz)

Figure S4. ^{13}C-NMR spectrum of compound **2** ($CDCl_3$, 125 MHz)

Figure S5. ^{1}H-NMR spectrum of compound **4** ($CDCl_3$, 500 MHz)

Figure S6. ^{13}C-NMR spectrum of compound **4** ($CDCl_3$, 125 MHz)

Figure S7. DEPT spectrum of compound **4** ($CDCl_3$, 125 MHz)

Figure S8. HSQC spectrum of compound **4** ($CDCl_3$, 500 MHz)

Figure S9. COSY spectrum of compound **4** (CDCl3, 500 MHz)

Figure S10. HBMC spectrum of compound **4** (CDCl3, 500 MHz)

Figure S11. EIMS spectrum of compound **4**

Figure S12. HREIMS of compound **4**

Figure S13. ^{1}H-NMR spectrum of compound **7** ($CDCl_3$, 500 MHz)

Figure S14. ^{13}C-NMR spectrum of compound **7** ($CDCl_3$, 125 MHz)

Figure S15. ^{1}H-NMR spectrum of compound **8** ($CDCl_3$, 500 MHz)

Figure S16. ^{13}C-NMR spectrum of compound **8** ($CDCl_3$, 125 MHz)

Figure S17. ^{1}H-NMR spectrum of compound **14** ($CDCl_3$, 500 MHz)

Figure S18. ^{13}C-NMR spectrum of compound **14** ($CDCl_3$, 125 MHz)

Figure S19. HSQC spectrum of compound **14** ($CDCl_3$, 500 MHz)

Figure S20. COSY spectrum of compound **14** ($CDCl_3$, 500 MHz)

Figure S21. HMBC spectrum of compound **14** ($CDCl_3$, 500 MHz)

Figure S22. NOESY spectrum of compound **14** (CDCl3, 500 MHz)

Figure S23. HRESIMS of compound **14**

Sequence data of fungus *Phyllosticta sp.*

>YCC4

TATCAATAAGCGGAGGAAAAGAAACCAACAGGGATTGCCTTAGTAACGGCGAGTGAAGCGGCAATAGCTC

AAATTTGAAAGCTGGCGTCTTCGACGTCCGCGTTGTAATTTGTAGAGGATGCTTCGGCGAAGACTCCTGC

CTAAGTCCCCTGGAACGGGGCGTCATAGAGGGTGAGAATCCCGTATGTGGCGGGCAGTCTAAGCCATGTG

AAGCTCCTTCGACGAGTCGAGTTGTTTGGGAATGCAGCTCTAAATGGGAGGTAAATTTCTTCTAAAGCTA

AATATTGGCCAGAGACCGATAGCGCACAAGTAGAGTGATCGAAAGATGAAAAGCACTTTGGAAAGAGAGT

TAAAAAGTACGTGAAATTGTTGAAAGGGAAGCGCTTGCAACCAGACTCGCTCGTAGTTGCTCAGCCGGCC

TCTTGGCCGGTGTACTCTTCTACGATCGGGCCAGCATCAGTTCGGGCGGCAGGATAAAGGTGTCGGGAAT

GTAGCACCCTTCGGGTGTGTTATAGCCCGGCGCGGAATGCTGCCAGCCTGGACTGAGGATCTCGCTTCGG

CAAGGATGCTGGCGTAATGGTTGTAAGCGGCCCGTCTTGAAACACGGA

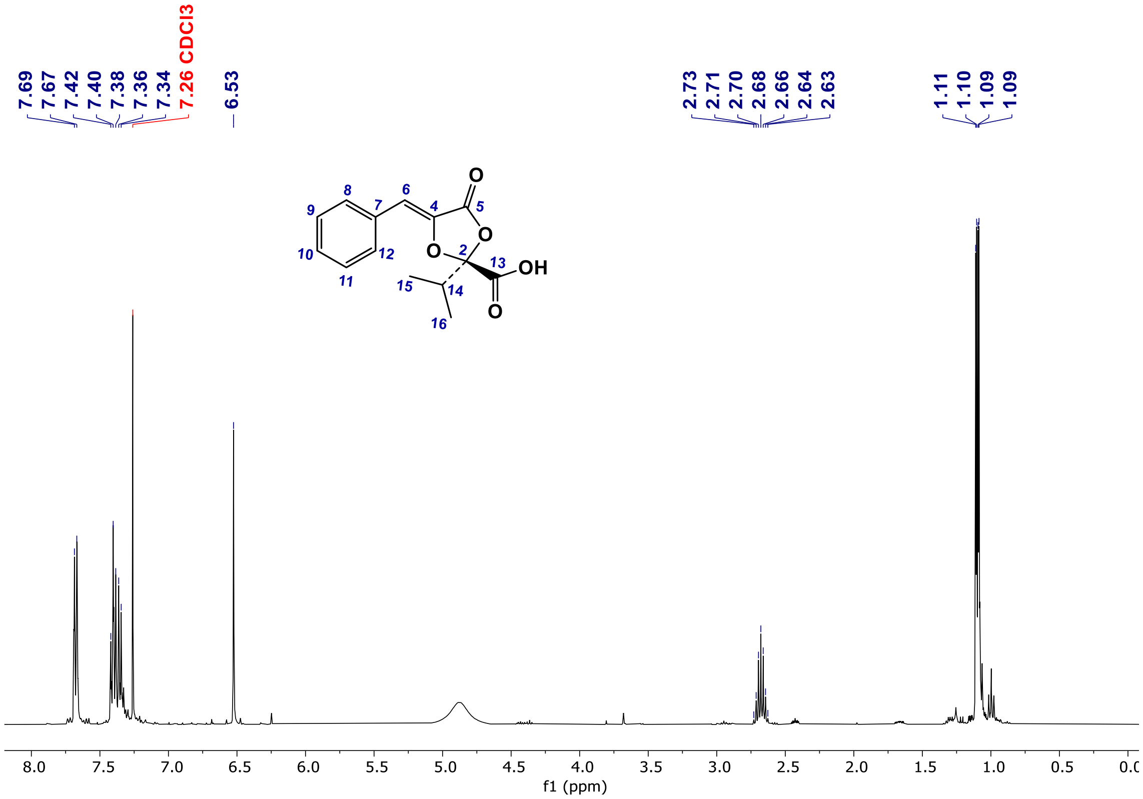

Figure S1. ^{1}H-NMR spectrum of compound **1** ($CDCl_3$, 500 MHz)

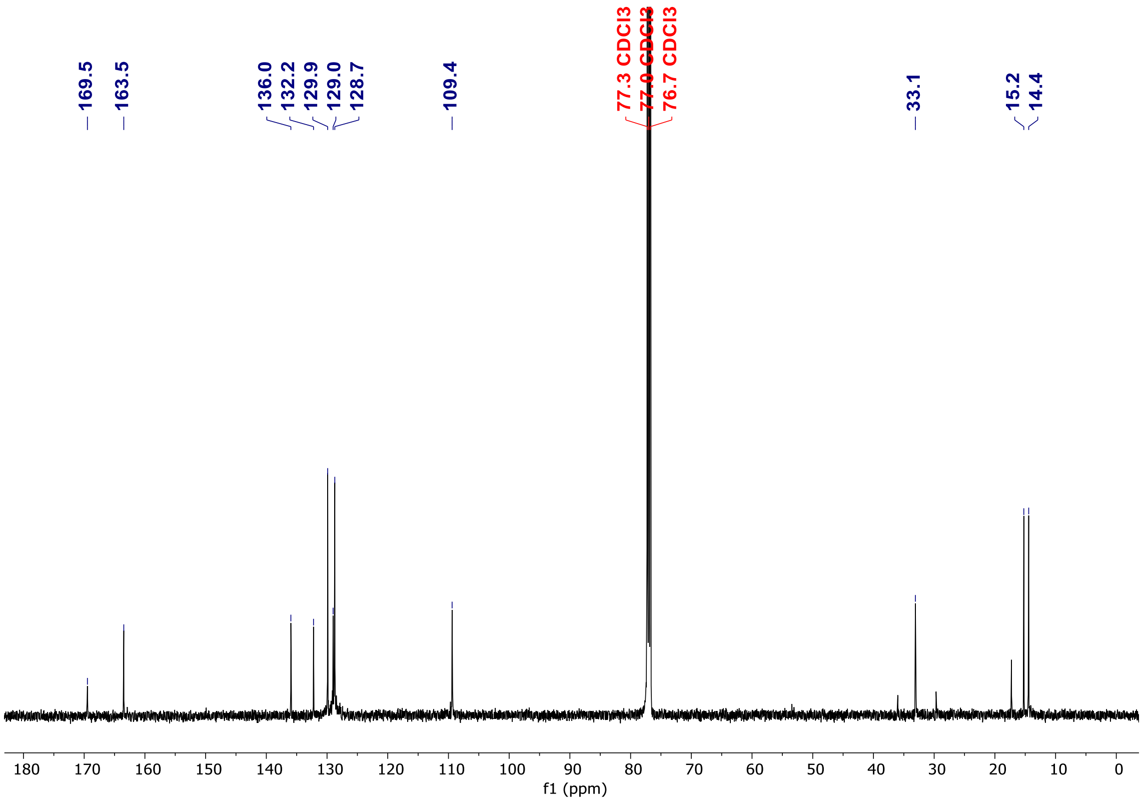

Figure S2. ^{13}C-NMR spectrum of compound **1** ($CDCl_3$, 125 MHz)

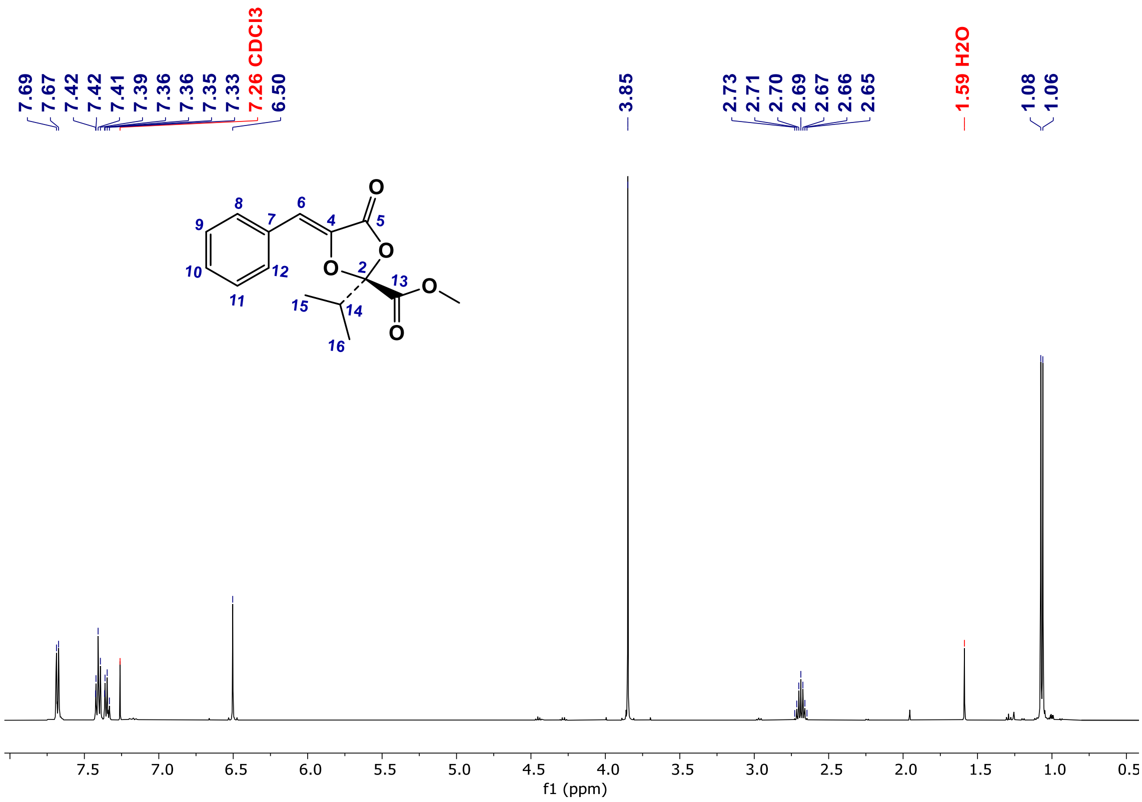

Figure S3. ^{1}H-NMR spectrum of compound **2** ($CDCl_3$, 500 MHz)

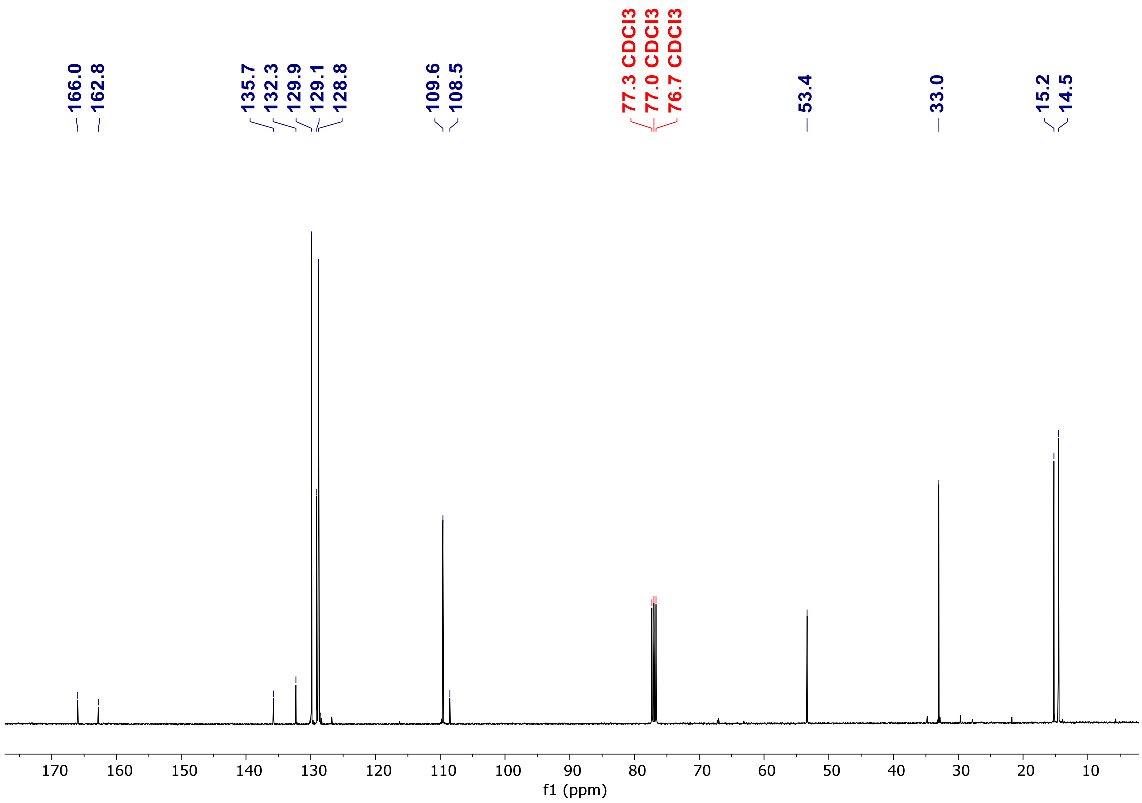

Figure S4. ^{13}C-NMR spectrum of compound **2** ($CDCl_3$, 125 MHz)

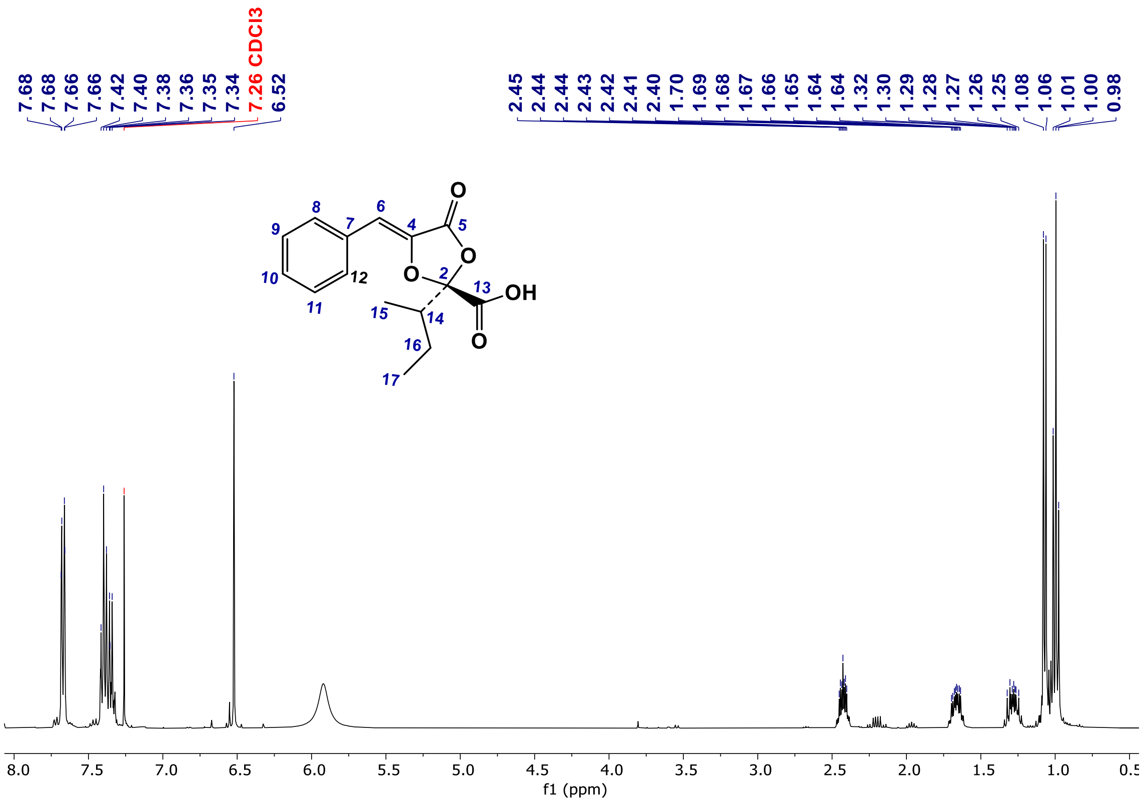

Figure S5. ^{1}H-NMR spectrum of compound **4** ($CDCl_3$, 500 MHz)

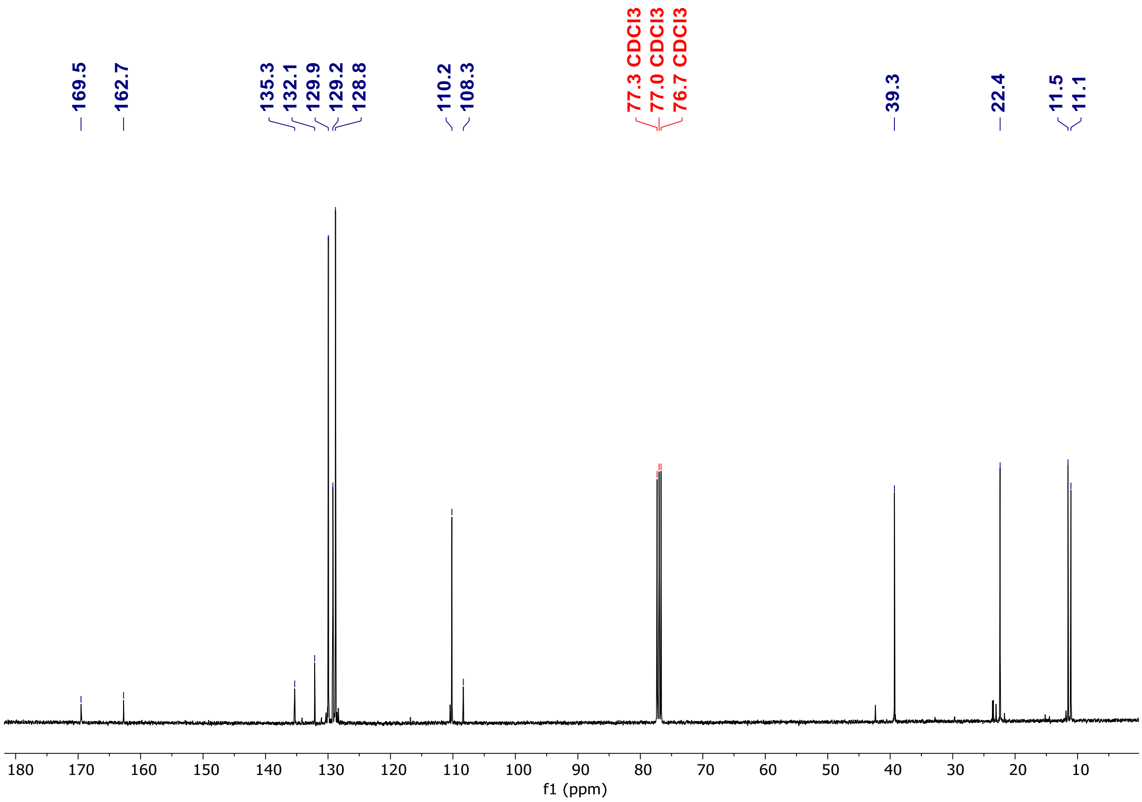

Figure S6. ^{13}C-NMR spectrum of compound **4** ($CDCl_3$, 125 MHz)

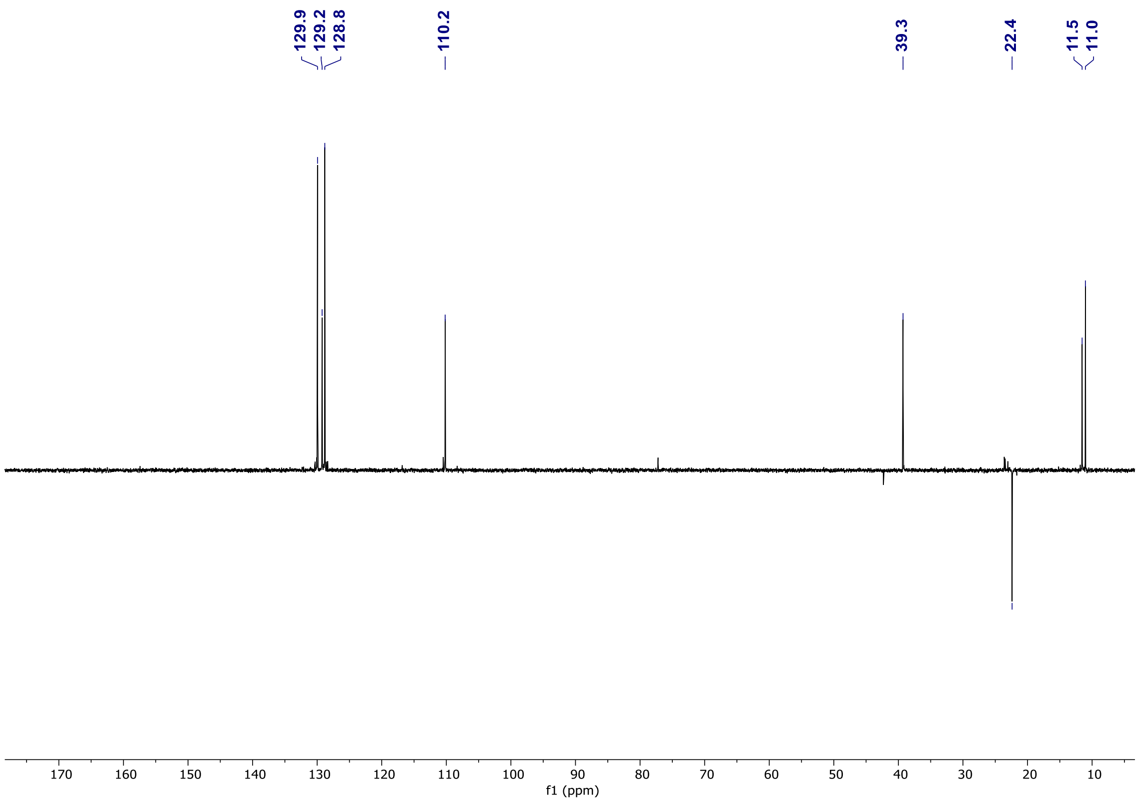

Figure S7. DEPT spectrum of compound **4** ($CDCl_3$, 125 MHz)

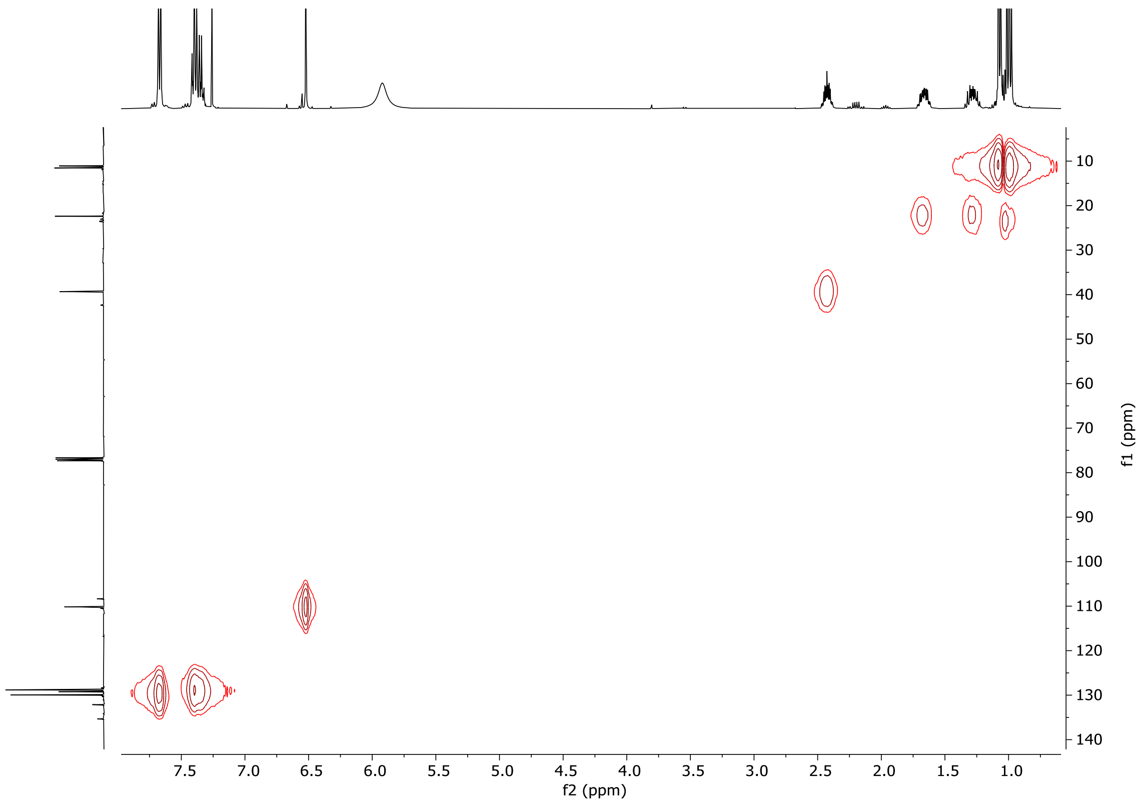

Figure S8. HSQC spectrum of compound **4** ($CDCl_3$, 500 MHz)

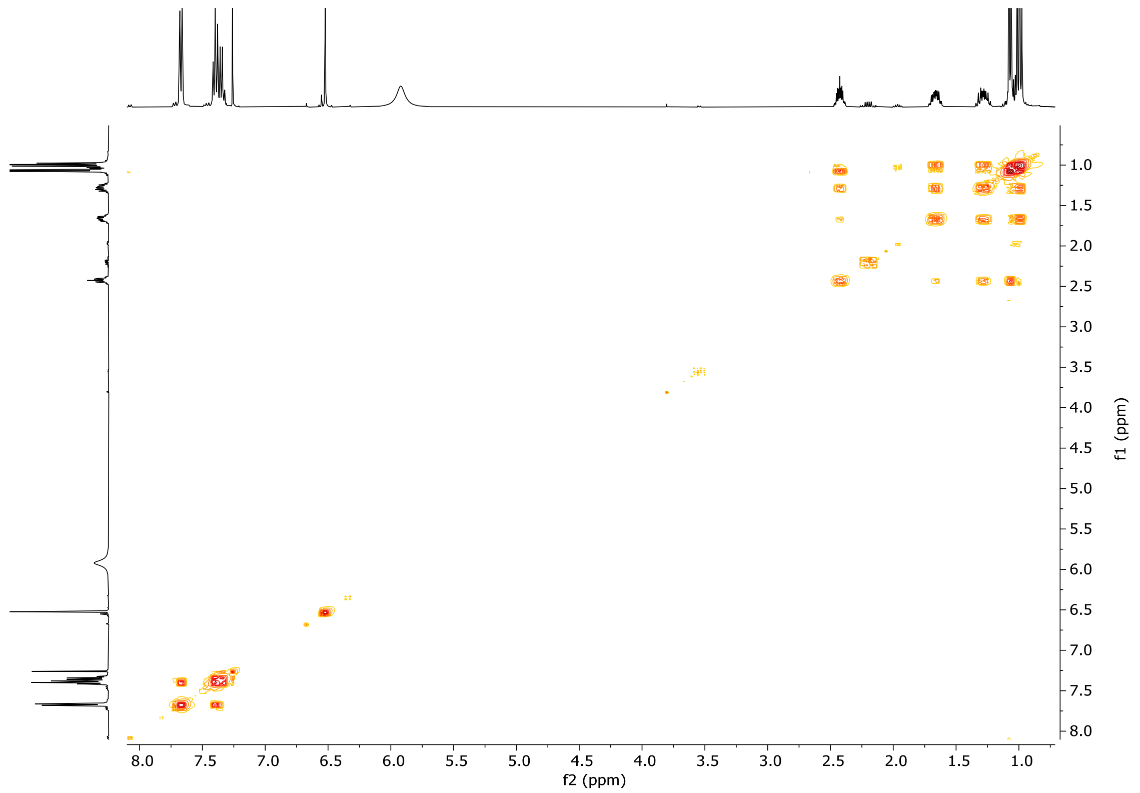

Figure S9. COSY spectrum of compound **4** ($CDCl_3$, 500 MHz)

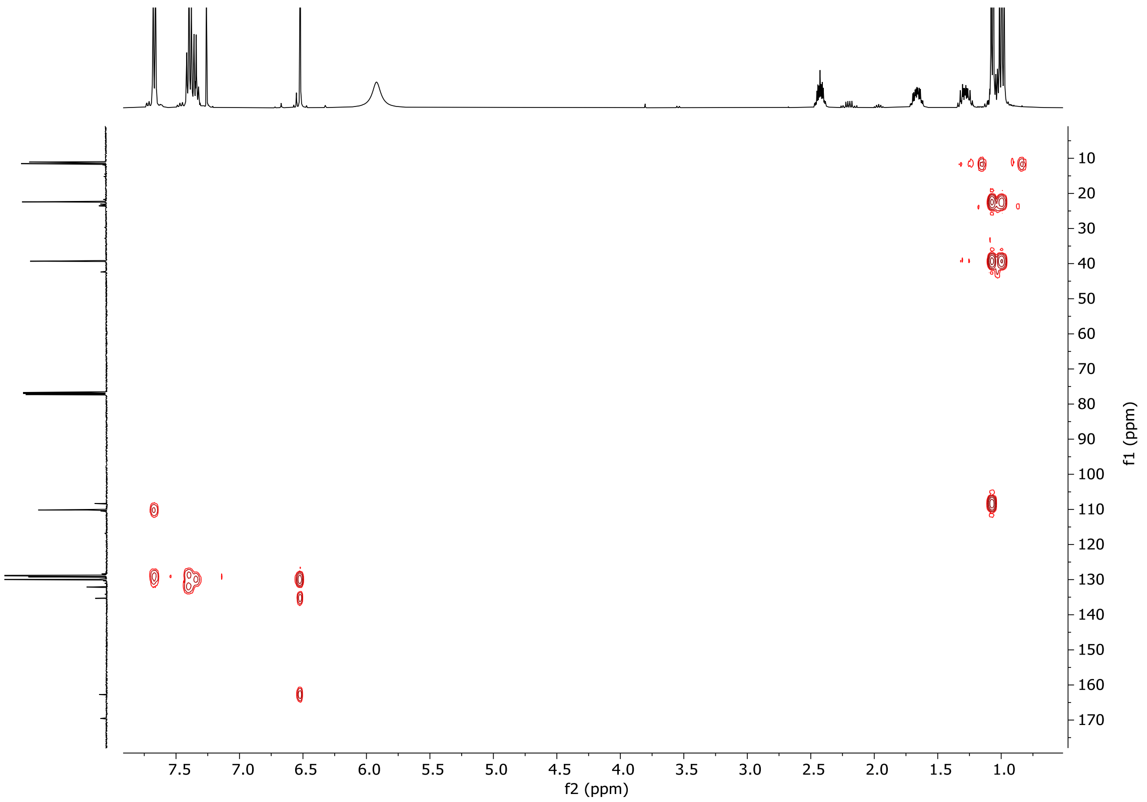

Figure S10. HMBC spectrum of compound **4** ($CDCl_3$, 500 MHz)

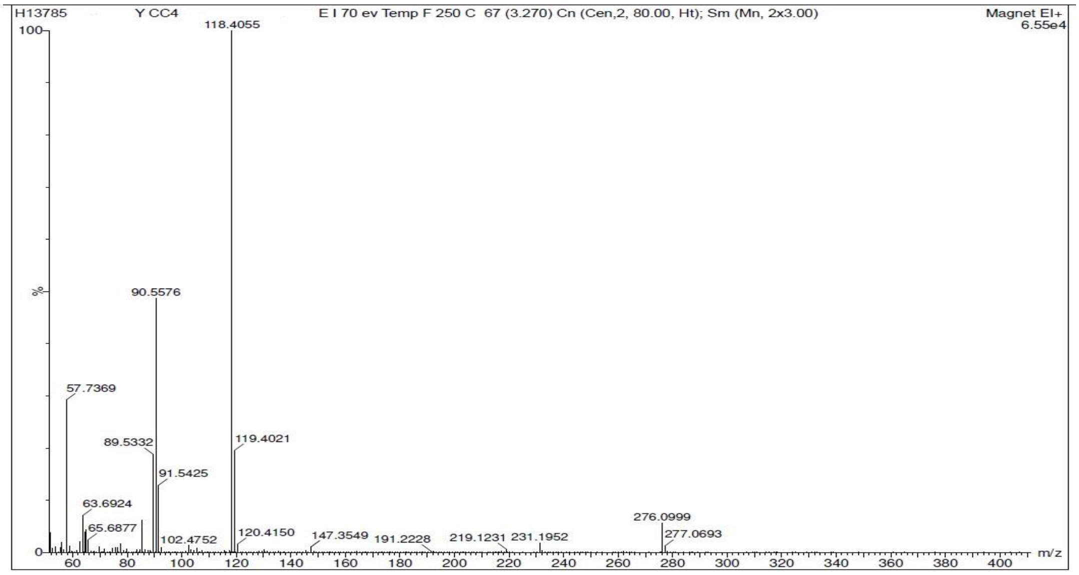

Figure S11. EIMS spectrum of compound **4**

Elemental Composition Report **Page 1**

Multiple Mass Analysis: 2 mass(es) processed
Tolerance = 5.0 PPM / DBE: min = -1.5, max = 200.0
Element prediction: Off
Number of isotope peaks used for i-FIT = 9

Monoisotopic Mass, Odd and Even Electron Ions
118 formula(e) evaluated with 1 results within limits (up to 2 closest results for each mass)
Elements Used:
C: 0-30 H: 0-40 O: 0-5 Na: 0-2
Patricia (Y CC4 Col 13 (10-1--10-3) E/I 70 ev Temp F 250 C
H13785-Patricia (Y CC4 Col 13 (10-1--10-3) E I 70 ev Temp F 250 C 67 (3.270) Cn (Cen,2, 80.00, Ht); Sm (Mn, 2x3.00) Magnet EI+ 3.72e+003

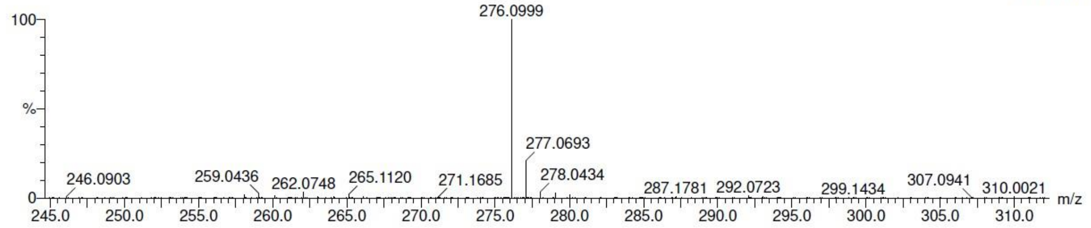

Minimum:	5.60				-1.5			
Maximum:	100.00		5.0	5.0	200.0			
Mass	RA	Calc. Mass	mDa	PPM	DBE	i-FIT	i-FIT (Norm)	Formula
276.0999	100.00	276.0998	0.1	0.4	8.0	157.1	0.0	C15 H16 O5

Figure S12. HREIMS of compound **4**

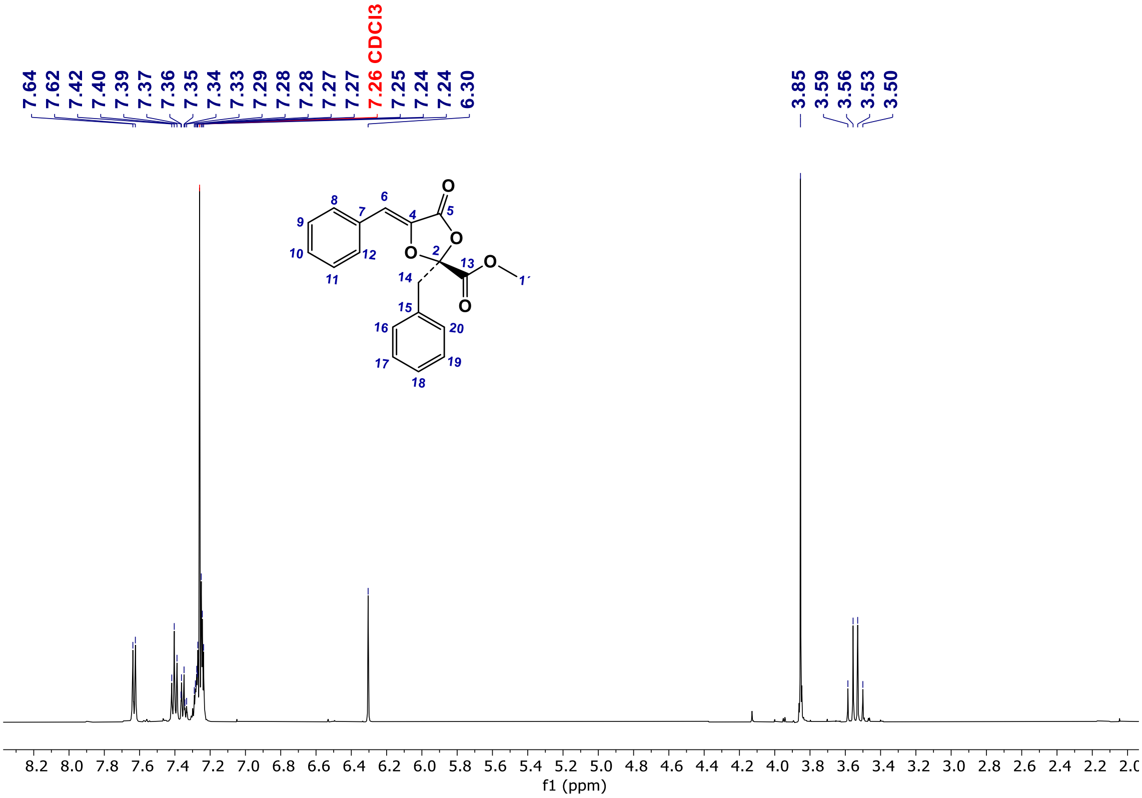

Figure S13. ^{1}H-NMR spectrum of compound **7** ($CDCl_3$, 500 MHz)

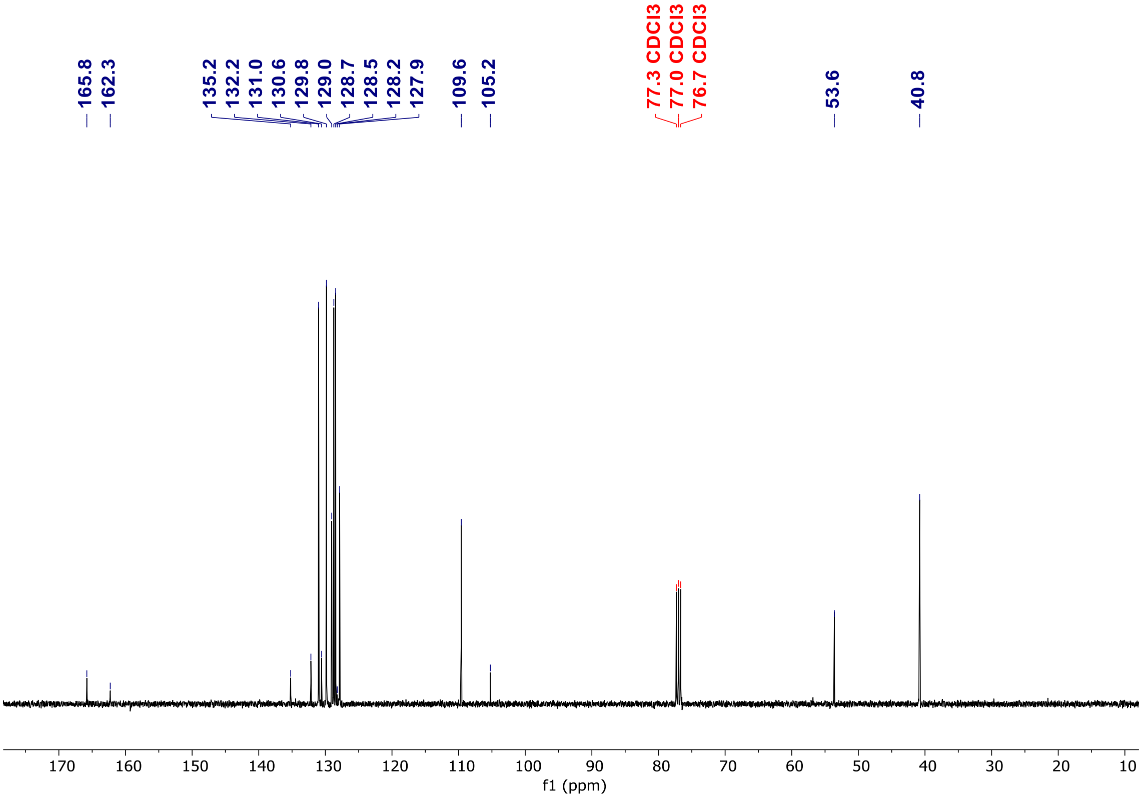

Figure S14. ^{13}C-NMR spectrum of compound **7** ($CDCl_3$, 125 MHz)

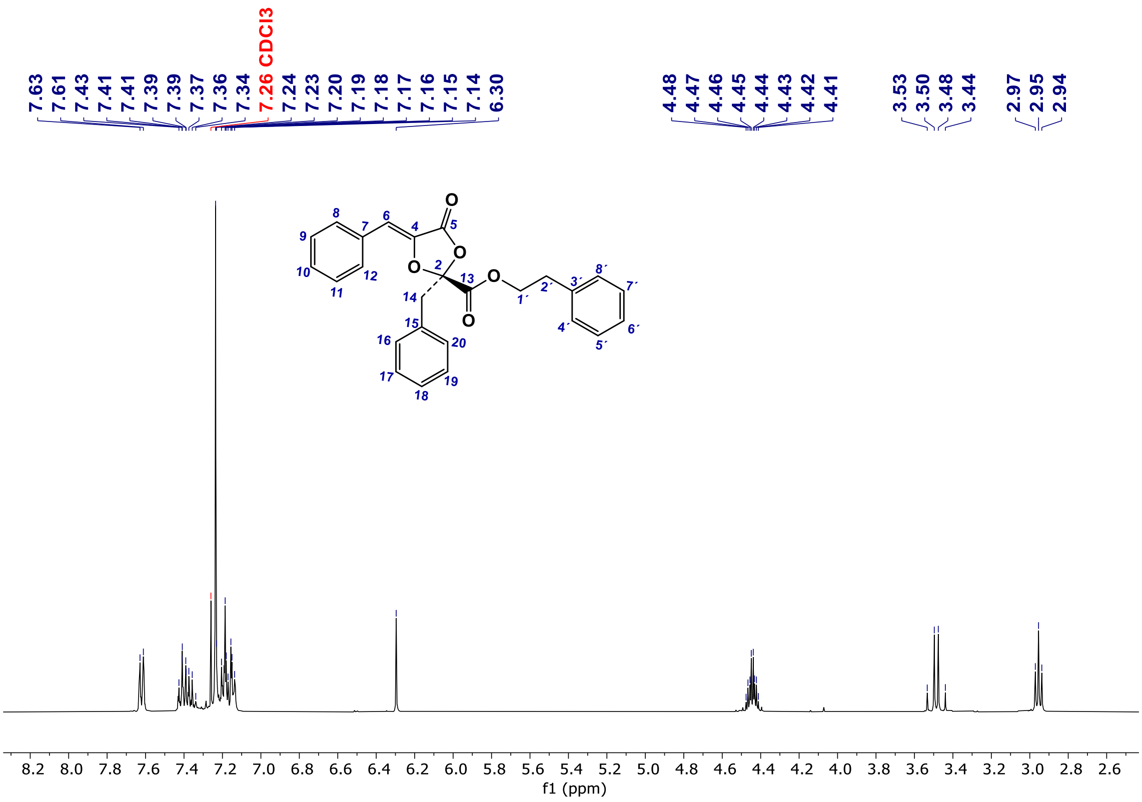

Figure S15. ^{1}H-NMR spectrum of compound **8** ($CDCl_3$, 500 MHz)

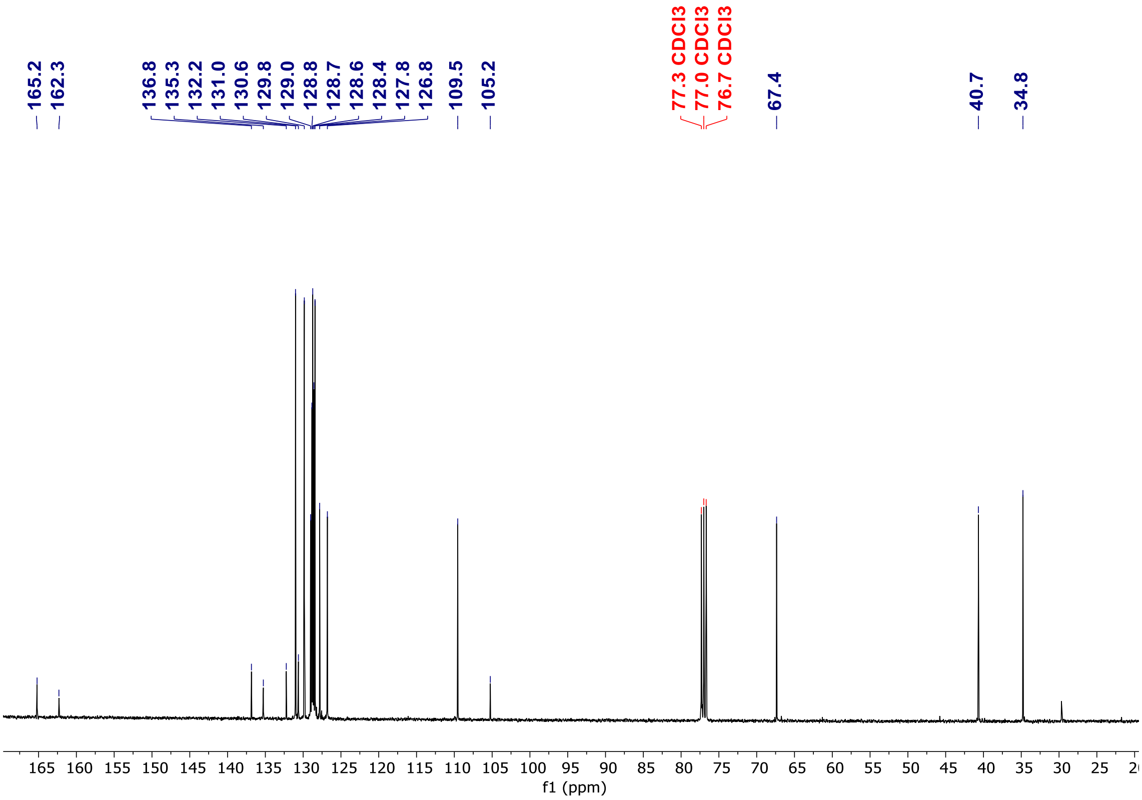

Figure S16. ^{13}C-NMR spectrum of compound **9** ($CDCl_3$, 125 MHz)

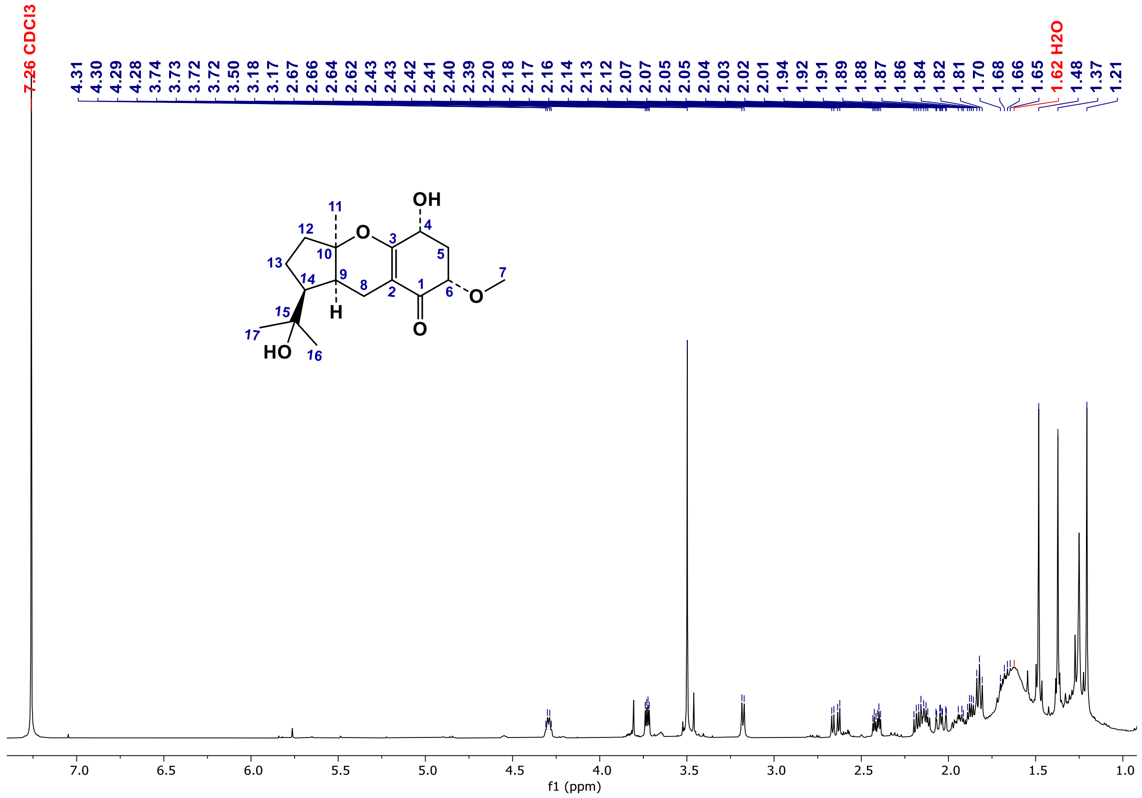

Figure S17. ^{1}H-NMR spectrum of compound **14** (CDCl3, 500 MHz)

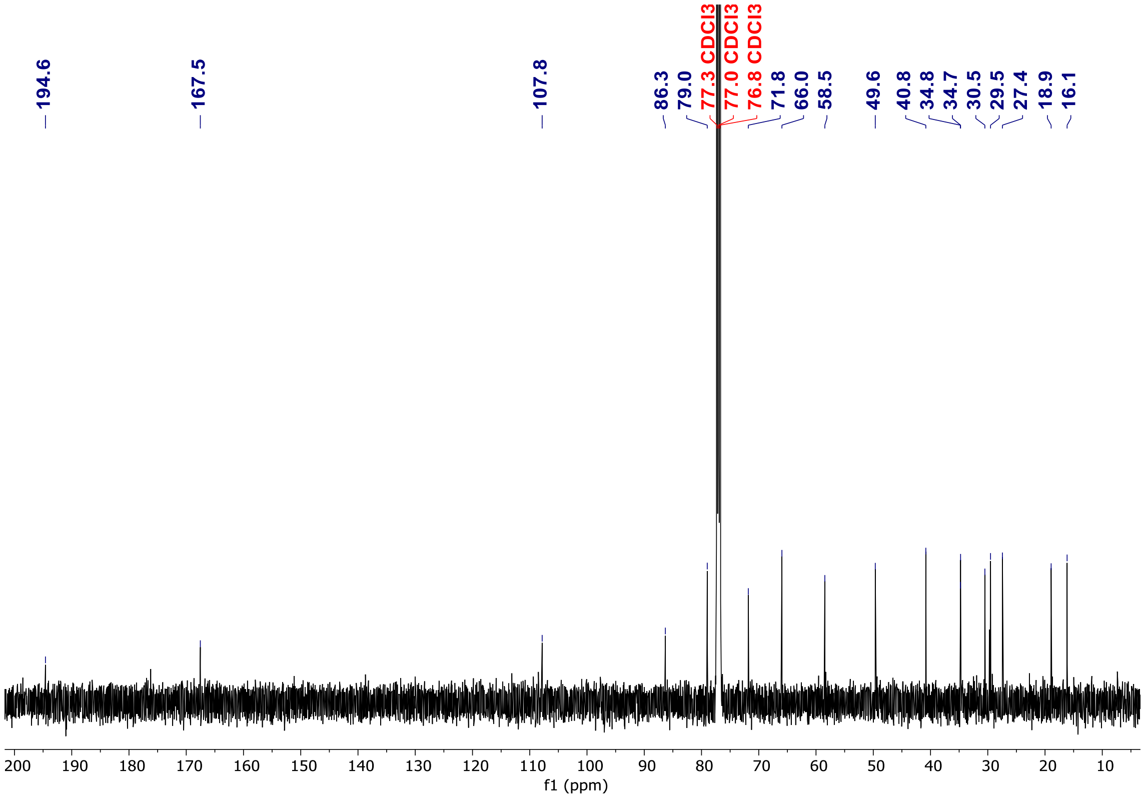

Figure S18. ^{13}C-NMR spectrum of compound **14** (CDCl3, 125 MHz)

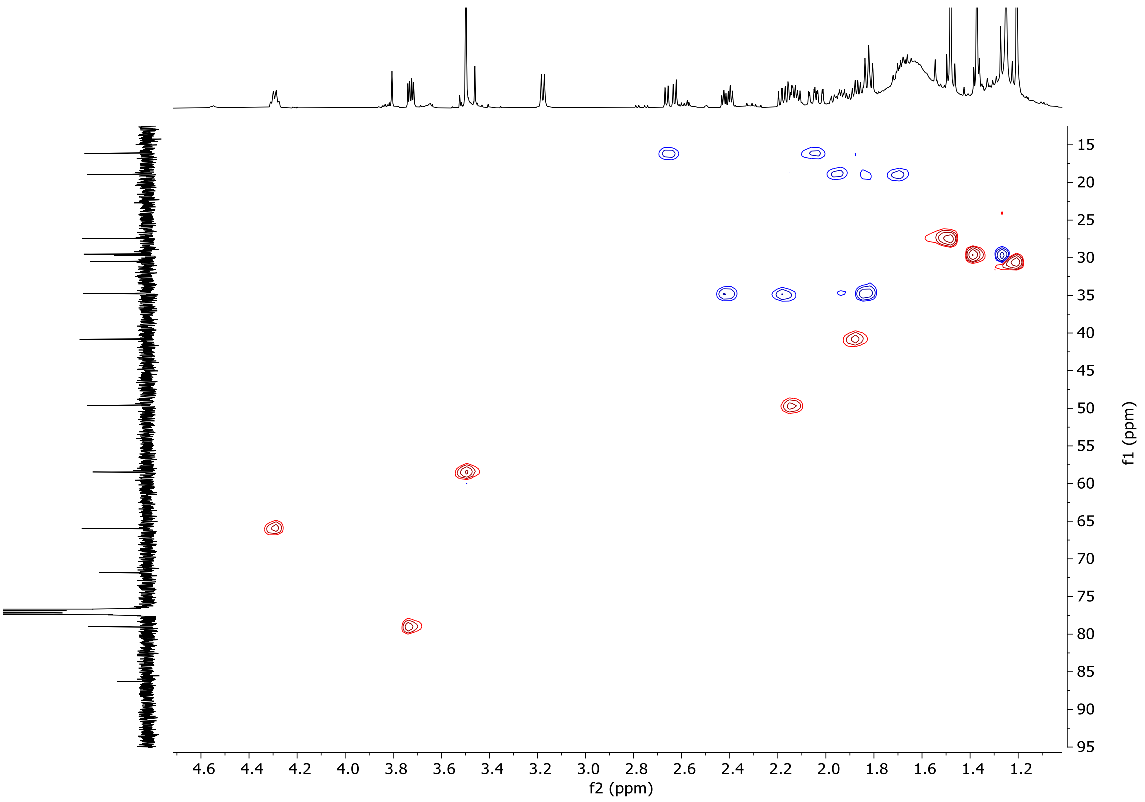

Figure S19. HSQC spectrum of compound **14** ($CDCl_3$, 500 MHz)

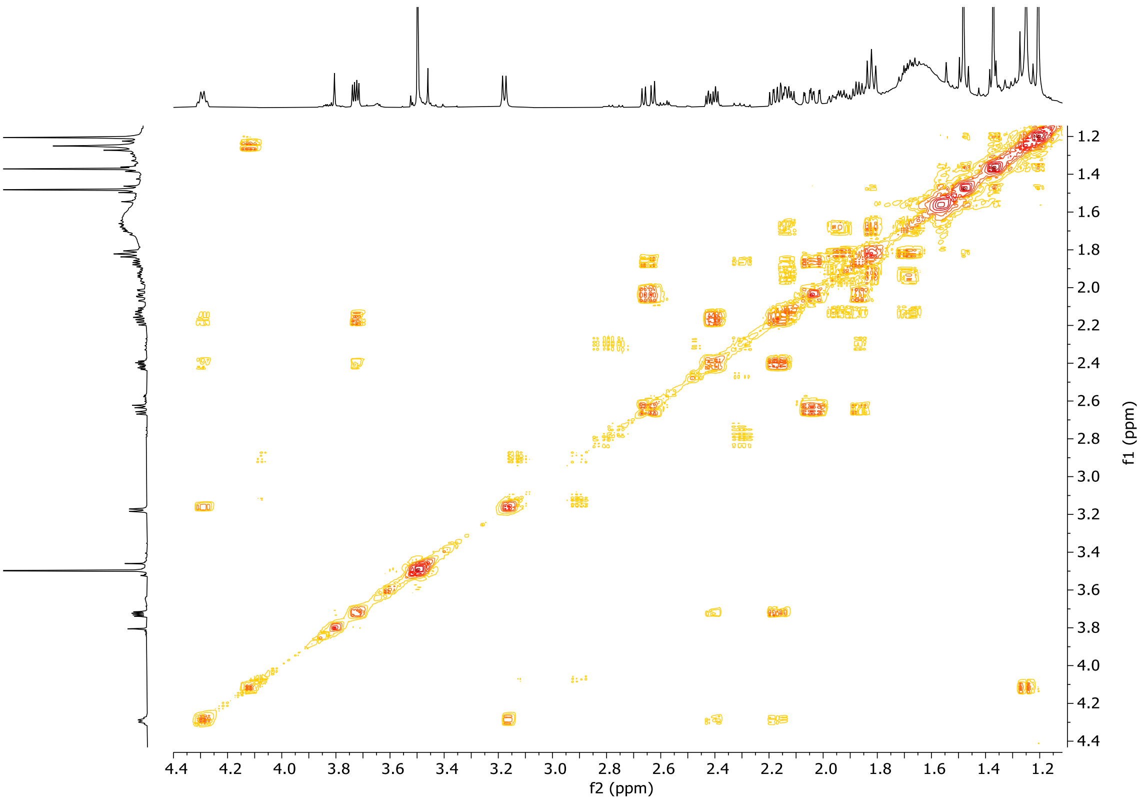

Figure S20. COSY spectrum of compound **14** ($CDCl_3$, 500 MHz)

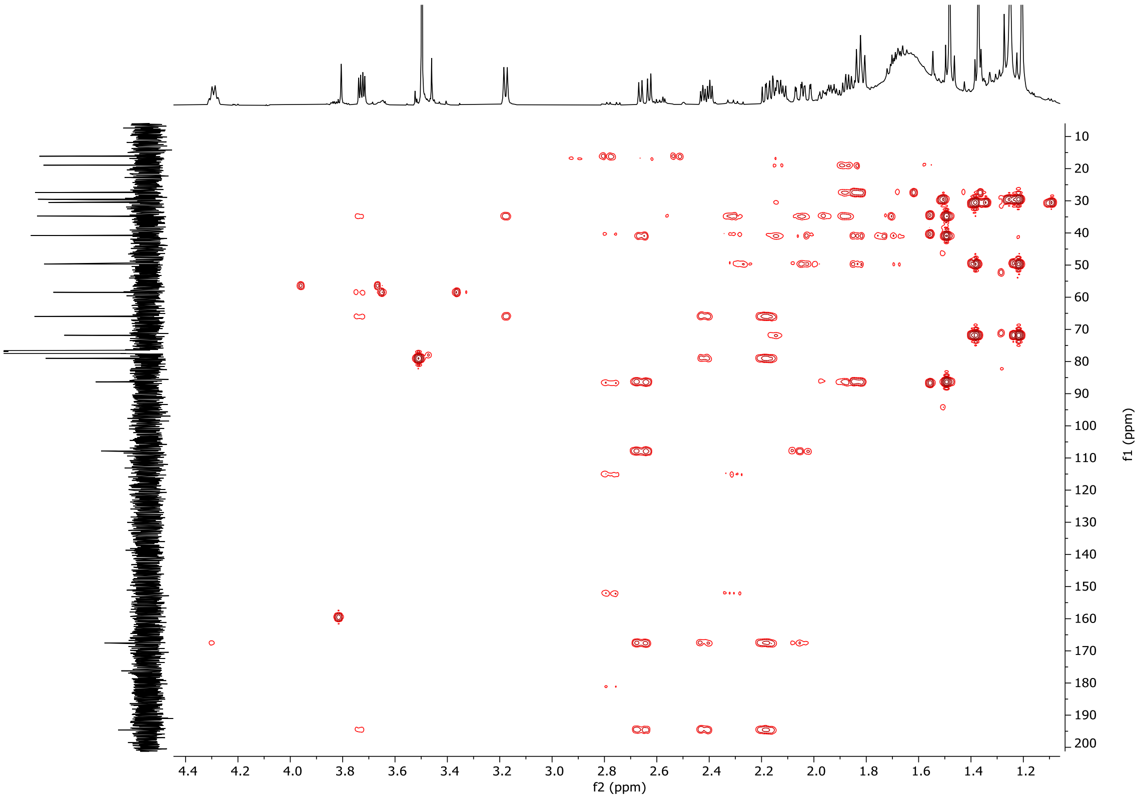

Figure S21. HMBC spectrum of compound **14** (CDCl3, 500 MHz)

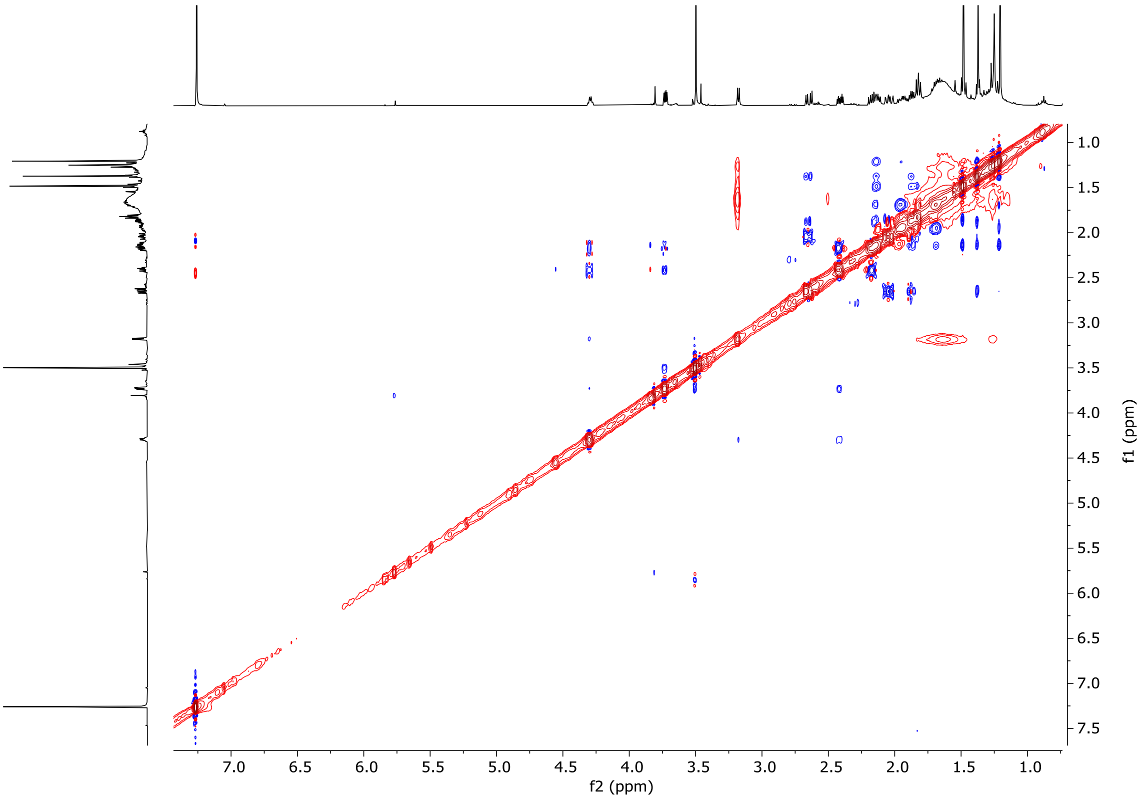

Figure S22. NOESY spectrum of compound **14** (CDCl3, 125 MHz)

Elemental Composition Report Page 1

Multiple Mass Analysis: 2 mass(es) processed
Tolerance = 5.0 PPM / DBE: min = -1.5, max = 200.0
Element prediction: Off
Number of isotope peaks used for i-FIT = 9

Monoisotopic Mass, Even Electron Ions
64 formula(e) evaluated with 4 results within limits (up to 2 closest results for each mass)
Elements Used:
C: 0-30 H: 0-40 N: 0-2 O: 5-5 Na: 0-2
Daniel
H 2998-Patricia (Y CC4 Col 44 25-6---26-4) 42 (1.463) 2: TOF MS ES+
1.23e+004

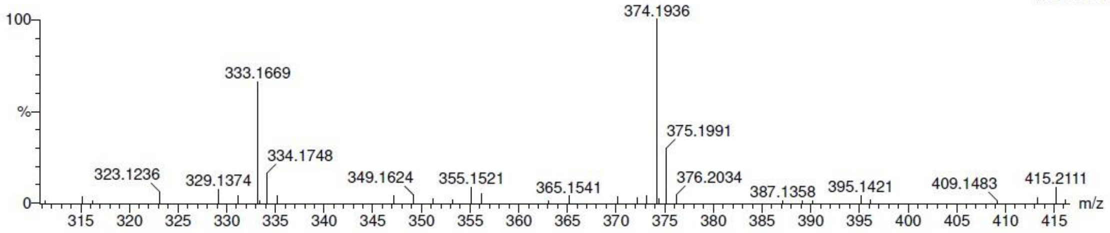

Mass	RA	Calc. Mass	mDa	PPM	DBE	i-FIT	i-FIT (Norm)	Formula
Minimum:	60.00				-1.5			
Maximum:	100.00		5.0	5.0	200.0			
333.1669	65.96	333.1678	-0.9	-2.7	4.5	53.4	0.7	C17 H26 O5 Na
		333.1654	1.5	4.5	1.5	53.4	0.7	C15 H27 O5 Na2
374.1936	100.00	374.1943	-0.7	-1.9	5.5	58.0	0.5	C19 H29 N O5 Na

Figure S23. HRESIMS of compound **14**
